# Visit Types in Primary Care With Telehealth Use During the COVID-19 Pandemic: Systematic Review

**DOI:** 10.2196/40469

**Published:** 2022-11-28

**Authors:** Kanesha Ward, Sanjyot Vagholkar, Fareeya Sakur, Neha Nafees Khatri, Annie Y S Lau

**Affiliations:** 1 Centre for Health Informatics Australian Institute for Health Innovation Macquarie University North Ryde Australia; 2 Primary Care Faculty of Medicine, Health & Human Sciences Macquarie University North Ryde Australia

**Keywords:** primary care, general practitioners, telehealth, telemedicine, COVID-19, remote consultation, video consultation, eHealth

## Abstract

**Background:**

Telehealth was rapidly incorporated into primary care during the COVID-19 pandemic. However, there is limited evidence on which primary care visits used telehealth.

**Objective:**

The objective of this study was to conduct a systematic review to assess what visit types in primary care with use of telehealth during the COVID-19 pandemic were reported; for each visit type identified in primary care, under what circumstances telehealth was suitable; and reported benefits and drawbacks of using telehealth in primary care during the COVID-19 pandemic.

**Methods:**

This study was a systematic review using narrative synthesis. Studies were obtained from four databases (Ovid [MEDLINE], CINAHL Complete, PDQ-Evidence, and ProQuest) and gray literature (NSW Health, Royal Australian College of General Practitioners guidelines, and World Health Organization guidelines). In total, 3 independent reviewers screened studies featuring telehealth use during the COVID-19 pandemic in primary care. Levels of evidence were assessed according to the Grading of Recommendations Assessment, Development, and Evaluation. Critical appraisal was conducted using the Mixed Methods Appraisal Tool. Benefits and drawbacks of telehealth were assessed according to the National Quality Forum Telehealth Framework.

**Results:**

A total of 19 studies, predominately cross-sectional surveys or interviews (13/19, 68%), were included. Seven primary care visit types were identified: *chronic condition management* (17/19, 89%), *existing patients* (17/19, 89%), *medication management* (17/19, 89%), *new patients* (16/19, 84%), *mental health/behavioral management* (15/19, 79%), *post–test result follow-up* (14/19, 74%), and *postdischarge follow-up* (7/19, 37%). Benefits and drawbacks of telehealth were reported across all visit types, with *chronic condition management* being one of the visits reporting the greatest *use* because of a pre-existing patient-provider relationship, established diagnosis, and lack of complex physical examinations. Both patients and clinicians reported benefits of telehealth, including improved convenience, focused discussions, and continuity of care despite social distancing. Reported drawbacks included technical barriers, impersonal interactions, and semi-established reimbursement models.

**Conclusions:**

Telehealth was used for different visit types during the COVID-19 pandemic in primary care, with most visits for *chronic condition management*, *existing patients*, and *medication management*. Further research is required to validate our findings and explore the long-term impact of hybrid models of care for different visit types in primary care.

**Trial Registration:**

PROSPERO CRD42022312202; https://tinyurl.com/5n82znf4

## Introduction

### Background

The COVID-19 pandemic has radically disrupted all aspects of health care, notably the rapid adaption of telehealth within routine care [1-3]. *Telehealth*, defined as telecommunications, videoconferencing, or other digital modes, is used to remotely deliver health-related services to patients [4,5]. Before the COVID-19 pandemic, telehealth provided convenience, specifically for patients living in rural or remote settings, but was not routinely used in health care settings [5]. Telehealth during the pandemic was used across many medical specialties such as internal medicine, psychiatry, preventative medicine, surgery, neurology, dermatology, pediatrics, and infectious diseases [6].

In particular, some general practitioners (GPs) and patients welcomed telehealth in primary care general practice settings during the pandemic. A survey conducted by the Royal Australian College of General Practitioners (RACGP) involving >420 Australian GPs saw 1 in 5 respondents report 61% to 80% of their patients requesting a telehealth consultation during the COVID-19 pandemic [7]. Some patients and GPs have advocated for the long-term use of telehealth beyond the COVID-19 pandemic, for example, in the form of hybrid models of care [1,7-9]. Several countries (eg, Australia, the United States, and the United Kingdom) have introduced long-term funding for telehealth in primary care because of the pandemic.

There is potential for telehealth in primary care in nonpandemic settings [1]. However, the current model of telehealth may not be fit to sustain the long-term delivery of primary care [2,10,11]. As the rapid adoption of telehealth and other forms of remote care is witnessed, its limitations need to be examined [10]. Most telehealth systems were rolled out rapidly without much research into the risks (eg, lack of patient choice, missed diagnoses, challenges to the patient-clinician relationship, and inequality experienced by those affected by the digital divide) [1,10,12]. Identifying which in-person encounters are *appropriate* to be supported by telehealth consultation is one of the critical questions facing today’s health care delivery.

A cross-sectional study conducted by Donaghy et al [13] explored the acceptability and suitability of telehealth for specific encounters, where they reported telehealth as suitable for a range of patient visit types and concerns such as prescription refills, discussion-based activities, nonsensitive test results, and patients with chronic conditions with established diagnoses. A systematic review by Shah and Badawy [14] evaluated the feasibility, accessibility, satisfaction, and treatment outcomes related to telehealth services among pediatric populations, with findings suggesting telehealth to be a suitable alternative to in-person care. A previous systematic review by Snoswell et al [15] aimed to synthesize literature on the clinical effectiveness of telehealth for specific medical conditions from 2010 to 2019. However, to our knowledge, this is the first systematic review to focus on what visit types in primary care are suitable for telehealth based on studies where data were collected during the COVID-19 pandemic.

### Objectives

The objective of this study was to conduct a systematic review to assess (1) what visit types in primary care with use of telehealth during the COVID-19 pandemic were reported; (2) for each visit type identified in primary care, under what circumstances telehealth was suitable; and (3) reported benefits and drawbacks of using telehealth in primary care during the COVID-19 pandemic.

## Methods

### Information Sources

This review is PRISMA (Preferred Reporting Items for Systematic Reviews and Meta-Analyses)-compliant. See Multimedia Appendix 1 for the completed checklist of PRISMA guidelines.

Our search included the following electronic databases: Ovid (MEDLINE), CINAHL Complete, PDQ-Evidence, and ProQuest. Gray literature sources included NSW Health publications, RACGP guidelines, and World Health Organization guidelines.

### Search Strategy

A modified population, exposure, and outcome [16] strategy was used, with *population* corresponding to primary care general practice clinicians and patients; *exposure* as the exposure to telehealth as a replacement of in-person consultation; and *outcomes* as benefits and drawbacks of telehealth, which are assessed according to the National Quality Forum (NQF) Telehealth Framework [17], namely, access to care, effectiveness, experience, and financial impact or cost. Clinical outcomes outside the scope outlined per the NQF telehealth measures were not analyzed in detail in this systematic review because of the lack of available data. However, clinical outcomes (eg, mental health status, shielding status, and number of examinations) were also extracted in Multimedia Appendix 2 [1,9,18-34] if they were available.

Individualized search strategies were formulated for each selected database with various Medical Subject Headings and searchable terms combined with Boolean operators. The complete search strategy is provided in Multimedia Appendix 3. An initial full search was conducted in March 2020. A final full search was conducted in August 2022. Conducting 2 searches ensured that the most recent and relevant literature was included in this systematic review analysis. Including both searches also reflects the rapid rate at which research is being conducted on telehealth services used in primary care settings following the COVID-19 pandemic.

### Eligibility Criteria

Eligibility criteria were developed to include studies (1) published between December 2019 and August 2022 to encompass the COVID-19 era, (2) that discussed GP-patient consultations delivered within a telehealth format, (3) that provided insight into the visit types in primary care where telehealth was used, and (4) that included outcome measures on patients’ or clinicians’ perceived suitability of or satisfaction with the teleconsultation experience. Studies featuring multiple health care settings may also be included based on the fact that only data from primary care clinicians or patients were used for this systematic review.

The exclusion criteria were (1) telehealth services that did not reflect a consultation format (ie, did not involve bidirectional communication between clinician and patient) within the primary care general practice setting (specialist consultations excluded), (2) studies where it was not explicit in what visit type in primary care was telehealth being used, and (3) studies not written in English. The complete eligibility criteria are provided in Multimedia Appendix 4.

### Article Selection Process

Initially, titles and abstracts of studies were retrieved using our search strategy and uploaded to an EndNote (Clarivate Analytics) library [35]. Duplicates were removed before uploading to the Rayyan software (Rayyan Systems Inc) [36] for titles and abstracts to be screened independently by three reviewers (KW, FS, and NNK). The full texts of the selected studies were assessed in greater detail by lead reviewer KW. Disagreements in article screening decisions were resolved through consensus.

### Data Extraction and Management

Data from the included studies were extracted using an adapted version of the Joanna Briggs Institute data abstraction form (Multimedia Appendix 5) [37]. Publication details, study design, participant demographics, primary care visit type, telehealth intervention, and outcome measures were extracted from the included studies. Benefits and drawbacks of telehealth were extracted as outcome measures, presented according to the NQF Telehealth Framework. The NQF Telehealth Framework addresses the assessment of whether telehealth specifically can be used to deliver quality care and related outcomes in comparison with in-person consultations [16]. Definitions of each outcome measure used in this framework—namely, *access to care*, *effectiveness*, *experience*, and *financial impact or cost—*are reported in Textbox 1 [17]. Only relevant statistics or narrative excerpts were extracted. Effect measures were quoted from individual studies with no further statistical comparison.

**Outcome measures and their definitions according to the National Quality Forum Telehealth Framework.**

**Definitions of outcome measures**

- Access to care: the ability to receive health services promptly and appropriately; consideration for accessibility to technology, living in rural and urban communities, living in medically underserved areas, access to appropriate health specialists, and provider capacity to provide care
- Effectiveness: the systematic, clinical, operational, and technical success or barriers of telehealth; considerations of the overall system and care coordination established, impact on health outcomes or quality, how clinically integrated telehealth is within the health center, and ability to record and transmit necessary data
- Experience: the usability and effect of telehealth on patients and providers with consideration of the appropriateness of services, increase in patients’ knowledge of care, patient compliance with care regimens, the difference in morbidity and mortality rates, patient safety, patient-centeredness, efficiency, diagnostic accuracy, ability to obtain actionable information, comfort, and satisfaction
- Financial impact or cost: potential cost savings or losses to patients, families, or providers regarding costs to access care, travel expenses, added value, and feasibility surrounding the technology involved

### Critical Appraisal of the Included Studies

One reviewer (KW) led the critical appraisal. The Mixed Methods Appraisal Tool was used to appraise study designs of qualitative, quantitative, and mixed methods studies [38]. The level of evidence was assessed according to the Grading of Recommendations Assessment, Development, and Evaluation (GRADE) [39]. Studies were not excluded based on outcomes of the critical appraisal; however, it was used to interpret findings. More details of the critical appraisal are provided in Multimedia Appendix 6 [39,40].

## Results

### Screening Process

**Figure 1:**
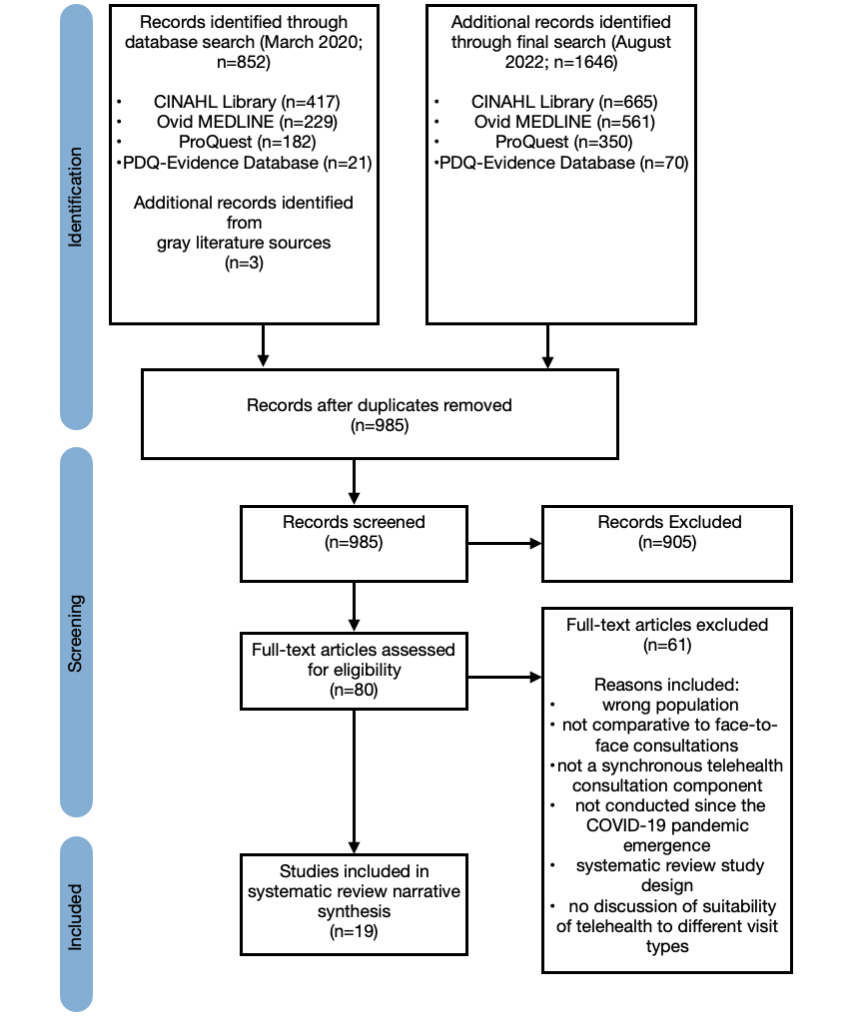
PRISMA (Preferred Reporting Items for Systematic Reviews and Meta-Analyses) flow diagram breakdown.

Figure 1 outlines the article screening process, where 19 studies met the eligibility criteria and were included in a narrative synthesis.

### Study Characteristics

Of the 19 included studies, 6 (32%) were conducted in the United States; 4 (21%) were conducted in the United Kingdom; 7 (37%) were conducted in Europe (Norway, Germany, Sweden, Netherlands, and Denmark); 3 (16%) were conducted in the Middle East (Israel and Oman); and the remaining 8 (42%) were conducted in Pakistan, Australia, and New Zealand (Multimedia Appendix 2).

Telephone communication (17/19, 89%) was the most frequent telehealth intervention in our included studies, followed by video communication (15/19, 79%), SMS text messaging (6/19, 32%), and email messaging (6/19, 32%). Table 1 provides a statistical breakdown of the types of telehealth interventions in the included studies.

**Table 1:** A statistical breakdown of the types of telehealth modes in the included studies (n=19).

Type of telehealth mode^a^	Studies, n (%)
Telephone communication	17 (89)
Video communication	15 (79)
SMS text messaging	6 (32)
Email messaging	6 (32)

^a^The included studies can discuss more than one telehealth mode.

### Visit Types in Primary Care With Telehealth Support During the COVID-19 Pandemic That Were Reported

Visit types in primary care with telehealth support during the COVID-19 pandemic that were reported are outlined in Textbox 2*.* Definitions of each visit type were informed by Medicare item descriptions (Multimedia Appendix 7 [29,41-43]) after extraction from the included studies.

Table 2 and Table 3 outline the reported benefits and drawbacks of using telehealth during the COVID-19 pandemic for each visit type in primary care. Seven visit types in primary care with telehealth use during the COVID-19 pandemic were reported, namely, *chronic condition management* (17/19, 89%), *existing patients* (17/19, 89%), *medication management* (17/19, 89%), *new patients* (16/19, 84%), *mental health/behavioral management* (15/19, 79%), *post–test result follow-up* (14/19, 74%), and *postdischarge follow-up* (7/19, 37%).

**Visit types in primary care with telehealth support during the COVID-19 pandemic that were reported [41]. Visit types do not categorize within age groups. Patient age is considered as a benefit or drawback finding for this review.**

**Visit types and description**

- Chronic condition management: 6-month or other routine chronic condition reviews, diabetes checkups, asthma or chronic obstructive pulmonary disease medication or management reviews, or chronic pain (ie, arthritis or musculoskeletal pain) discussions
- Mental health and behavioral management: anxiety, depression, behavioral treatment reviews, talking therapy, or mental health medication reviews; specialist visits excluded from this review
- Medication management: acute concerns (ie, antibiotics), medication reviews, oral contraceptive prescriptions, or dermatology prescriptions
- Post–test result follow-up: follow-up after magnetic resonance imaging examinations, x-rays, blood tests, or laboratory testing with their general practitioner (GP) to discuss given results
- Postdischarge follow-up: follow-up after a procedure or discharge from the hospital for patients with cancer after tumor removals, hospital admission following acute severe adverse reaction, or after pregnancy delivery
- Existing patients (acute or existing concerns): standard consultations with an annual checkup session or acute concerns (ie, cold or flu symptoms or dermatology concerns) with a patient the GP has a pre-existing patient-provider relationship; inclusive of patients with COVID-19 or shielding patients
- New patients (acute or existing concerns): standard consultations such as one-off sessions (eg, vaccination) or acute concerns (ie, cold or flu symptoms or dermatology concerns) with a patient with whom the GP has no pre-existing patient-provider relationship; inclusive of patients with COVID-19 or shielding patients

**Table 2:** Reported general practitioner-patient visit types with telehealth support during the COVID-19 pandemic (N=19).^a^

Visit type	Studies that reported the use of telehealth	Benefit findings of telehealth					
		Studies, n (%)	Level 1	Level 2	Level 3	Level 4	Level 5
Chronic condition management (n=17)	Johnsen et al [1] De Guzman et al [9] Jetty et al [18] Jabbarpour et al [20] Van de Poll-Franse et al [21] Gomez et al [22] Hasani et al [23] Imlach et al [24] Gabrielsson-Jarhult et al [25] Murphy et al [26] Schweiberger et al [27] RACGP^b^ [28] MBS^c^ [29] Mozes et al [30] Javanparast et al [31] Assing Hvidt et al [32] Due et al [33]	13 (76)	N/A^d^	N/A	Murphy et al [26]	Johnsen et al [1] De Guzman et al [9] Jetty et al [18] Jabbarpour et al [20] Gomez et al [22] Hasani et al [23] Schweiberger et al [27] Javanparast et al [31] Assing Hvidt et al [32] Due et al [33]	RACGP [28] MBS [29]
Medication management (nonchronic condition; n=17)	Johnsen et al [1] De Guzman et al [9] Jetty et al [18] Jabbarpour et al [20] Van de Poll-Franse et al [21] Gomez et al [22] Hasani et al [23] Imlach et al [24] Gabrielsson-Jarhult et al [25] Murphy et al [26] Schweiberger et al [27] RACGP [28] MBS [29] Mozes et al [30] Javanparast et al [31] Assing Hvidt et al [32] Due et al [33]	11 (65)	N/A	N/A	Gabrielsson-Jarhult et al [25]	Johnsen et al [1] De Guzman et al [9] Jetty et al [18] Gomez et al [22] Hasani et al [23] MBS [29] Mozes et al [30] Due et al [33]	RACGP [28] MBS [29]
Existing patients (acute or existing concern; n=17)	Johnsen et al [1] De Guzman et al [9] Jetty et al [18] Grossman et al [19] Jabbarpour et al [20] Van de Poll-Franse et al [21] Gomez et al [22] Hasani et al [23] Imlach et al [24] Murphy et al [26] Schweiberger et al [27] RACGP [28] MBS [29] Mozes et al [30] Javanparast et al [31] Assing Hvidt et al [32] Manski-Nankervis et al [34]	11 (65)	N/A	N/A	Imlach et al [24] Murphy et al [26] Mozes et al [30]	Johnsen et al [1] De Guzman et al [9] Grossman et al [19] Hasani et al [23] Schweiberger et al [27] RACGP [28] MBS [29] Assing Hvidt et al [32]	—^e^
New patients (acute or existing concern; n=16)	Johnsen et al [1] De Guzman et al [9] Jetty et al [18] Grossman et al [19] Jabbarpour et al [20] Van de Poll-Franse et al [21] Gomez et al [22] Hasani et al [23] Imlach et al [24] Gabrielsson-Jarhult et al [25] Schweiberger et al [27] RACGP [28] MBS [29] Assing Hvidt et al [32] Due et al [33] Manski-Nankervis et al [34]	7 (44)	N/A	N/A	Gabrielsson-Jarhult et al [25]	Johnsen et al [1] Hasani et al [23] Schweiberger et al [27] Assing Hvidt et al [32] Due et al [33]	MBS [29]
Mental health and behavioral management (n=15)	Johnsen et al [1] De Guzman et al [9] Jetty et al [18] Jabbarpour et al [20] Gomez et al [22] Hasani et al [23] Imlach et al [24] Murphy et al [26] Schweiberger et al [27] RACGP [28] MBS [29] Javanparast et al [31] Assing Hvidt et al [32] Due et al [33] Manski-Nankervis et al [34]	11 (73)	N/A	N/A	Imlach et al [24] Murphy et al [26]	Johnsen et al [1] De Guzman et al [9] Jabbarpour et al [20] Hasani et al [23] Schweiberger et al [27] Assing Hvidt et al [32] Due et al [33]	RACGP [28] MBS [29]
Post–test result follow-up (n=14)	Johnsen et al [1] De Guzman et al [9] Jetty et al [18] Jabbarpour et al [20] Van de Poll-Franse et al [21] Gomez et al [22] Hasani et al [23] Imlach et al [24] Murphy et al [26] Schweiberger et al [27] RACGP [28] MBS [29] Assing Hvidt et al [32] Due et al [33]	5 (36)	N/A	N/A	—	Johnsen et al [9] Gomez et al [22] Hasani et al [23] Assing Hvidt et al [32] Due et al [33]	—
Postdischarge follow-up (n=7)	Johnsen et al [1] Jetty et al [18] Hasani et al [23] Imlach et al [24] Murphy et al [26] Mozes et al [30] Javanparast et al [31]	5 (71)	N/A	N/A	Murphy et al [26]	Hasani et al [23]	RACGP [28] MBS [29]

^a^Definitions of the different visit types are informed by the Department of Health Medicare Benefits Scheme item definitions [41] (Multimedia Appendix 7). Levels of evidence were derived from the Grading of Recommendations Assessment, Development, and Evaluation scoring [39]. Level 1 is systematic reviews, level 2 is randomized controlled trials, level 3 is nonrandomized experimental studies or comparative (observational) studies, level 4 is case series (cohort studies), and level 5 is opinion pieces or clinical guidelines. Each article can report more than one visit type supported with telehealth during the COVID-19 pandemic.

^b^RACGP: Royal Australian College of General Practitioners.

^c^MBS: Medicare Benefits Schedule.

^d^N/A: not applicable.

^e^No data available for the category specified.

**Table 3:** Reported general practitioner-patient visit types with drawback findings of telehealth during the COVID-19 pandemic (N=19).^a^

Visit type	Studies that reported the use of telehealth	Drawback findings of telehealth					
		Studies, n (%)	Level 1	Level 2	Level 3	Level 4	Level 5
Chronic condition management (n=17)	Johnsen et al [1] De Guzman et al [9] Jetty et al [18] Jabbarpour et al [20] Van de Poll-Franse et al [21] Gomez et al [22] Hasani et al [23] Imlach et al [24] Gabrielsson-Jarhult et al [25] Murphy et al [26] Schweiberger et al [27] RACGP^b^ [28] MBS^c^ [29] Mozes et al [30] Javanparast et al [31] Assing Hvidt et al [32] Due et al [33]	6 (35)	N/A^d^	N/A	Gabrielsson-Jarhult et al [25] Mozes et al [30]	Johnsen et al [1] De Guzman et al [9] Van de Poll-Franse et al [21] Due et al [33]	—^e^
Medication management (nonchronic condition; n=17)	Johnsen et al [1] De Guzman et al [9] Jetty et al [18] Jabbarpour et al [20] Van de Poll-Franse et al [21] Gomez et al [22] Hasani et al [23] Imlach et al [24] Gabrielsson-Jarhult et al [25] Murphy et al [26] Schweiberger et al [27] RACGP [28] MBS [29] Mozes et al [30] Javanparast et al [31] Assing Hvidt et al [32] Due et al [33]	3 (18)	N/A	N/A	Imlach et al [24] Mozes et al [30]	Johnsen et al [1]	—
Existing patients (acute or existing concern; n=17)	Johnsen et al [1] De Guzman et al [9] Jetty et al [18] Grossman et al [19] Jabbarpour et al [20] Van de Poll-Franse et al [21] Gomez et al [22] Hasani et al [23] Imlach et al [24] Murphy et al [26] Schweiberger et al [27] RACGP [28] MBS [29] Mozes et al [30] Javanparast et al [31] Assing Hvidt et al [32] Manski-Nankervis et al [34]	1 (6)	N/A	N/A	—	De Guzman et al [9]	—
New patients (acute or existing concern; n=16)	Johnsen et al [1] De Guzman et al [9] Jetty et al [18] Grossman et al [19] Jabbarpour et al [20] Van de Poll-Franse et al [21] Gomez et al [22] Hasani et al [23] Imlach et al [24] Gabrielsson-Jarhult et al [25] Schweiberger et al [27] RACGP [28] MBS [29] Assing Hvidt et al [32] Due et al [33] Manski-Nankervis et al [34]	9 (56)	N/A	N/A	—	Johnsen et al [1] De Guzman et al [9] Gomez et al [22] Hasani et al [23] Gabrielsson-Jarhult et al [25] Assing Hvidt et al [32] Due et al [33]	RACGP [28] MBS [29]
Mental health and behavioral management (n=15)	Johnsen et al [1] De Guzman et al [9] Jetty et al [18] Jabbarpour et al [20] Gomez et al [22] Hasani et al [23] Imlach et al [24] Murphy et al [26] Schweiberger et al [27] RACGP [28] MBS [29] Javanparast et al [31] Assing Hvidt et al [32] Due et al [33] Manski-Nankervis et al [34]	3 (20)	N/A	N/A	—	De Guzman et al [9] Due et al [33] Manski-Nankervis et al [34]	—
Post–test result follow-up (n=14)	Johnsen et al [1] De Guzman et al [9] Jetty et al [18] Jabbarpour et al [20] Van de Poll-Franse et al [21] Gomez et al [22] Hasani et al [23] Imlach et al [24] Murphy et al [26] Schweiberger et al [27] RACGP [28] MBS [29] Assing Hvidt et al [32] Due et al [33]	3 (21)	N/A	N/A	—	Jetty et al [18] Hasani et al [23] Due et al [33]	—
Postdischarge follow-up (n=7)	Johnsen et al [1] Jetty et al [18] Hasani et al [23] Imlach et al [24] Murphy et al [26] Mozes et al [30] Javanparast et al [31]	3 (43)	N/A	N/A	—	Johnsen et al [1] Jetty et al [18] Hasani et al [23]	—

^a^Definitions of the different visit types are informed by the Department of Health Medicare Benefits Scheme item definitions [41] (Multimedia Appendix 7). Levels of evidence were derived from the Grading of Recommendations Assessment, Development, and Evaluation scoring [39]. Level 1 is systematic reviews, level 2 is randomized controlled trials, level 3 is nonrandomized experimental studies or comparative (observational) studies, level 4 is case series (cohort studies), and level 5 is opinion pieces or clinical guidelines. Each article can report more than one visit type supported with telehealth during the COVID-19 pandemic.

^b^RACGP: Royal Australian College of General Practitioners.

^c^MBS: Medicare Benefits Schedule.

^d^N/A: not applicable.

^e^No data available for the category specified.

The benefits and drawbacks of using telehealth during the COVID-19 pandemic in primary care were reported across all visit types. Visit types with >60% of studies reporting benefits included *chronic condition management*, *mental health/behavioral management*, *medication management*, and *existing patients*, whereas the visit types with 40% of studies reporting drawbacks of telehealth included *new patients* and *postdischarge follow-up*.

Diverse study designs according to GRADE were reported in the included studies, with most (13/19, 68%) corresponding to level-4 evidence (cohort studies, interviews, and surveys), followed by level 3 (nonrandomized experimental studies or comparative or observational studies; 4/19, 21%) and level 5 (opinion pieces or clinical guidelines; 2/19, 11%). No randomized controlled trials (level-2 evidence) or systematic reviews (level-1 evidence) were found to have met the eligibility criteria to be included in this systematic review.

### Suitability of Using Telehealth Support for Each Visit Type During the COVID-19 Pandemic

#### Overview

For each visit type in primary care during the COVID-19 pandemic where telehealth support was reported, benefits and drawbacks are outlined in this section. Table 4 provides a summary of the circumstances when telehealth was reported as suitable and not suitable per patient visit types during the COVID-19 pandemic. For more details on supporting evidence, please refer to Multimedia Appendix 8 [1,9,20-26,28-34, 39,40,44-48].

**Table 4:** Circumstances when telehealth was reported as suitable and not suitable per patient visit types during the COVID-19 pandemic.

Visit type and subcategory		Circumstances when telehealth was suitable	Circumstances when telehealth was NOT suitable
**Condition- or concern-based**			
	Chronic condition management	Pre-existing patient-provider relationship [1,23,27] Established diagnosis [18] Lack of complex physical examinations [20]	Chronic conditions when there were complex issues requiring close monitoring or longer consultations (eg, complex comorbidities, cancer, complex social issues, low hearing and vision, and cognitive impairment) [1,9,25,30]
	Medication (nonchronic condition) management	Prescription refills of existing medications [1,9,22,32] Simple, straightforward health concerns (eg, oral contraceptives) [1,22] Predominately discussion-based activities [1,22]	When physical examinations were necessary (eg, prescribing antibiotics) [1,24,31] Prescription of new medications [1,24]
	Mental health and behavioral management	Patients with mild mental health issues (ie, not at risk to themselves or others or without high cognitive impairments) [20] Patients who did not prefer a physical presence [9,20] Predominately discussion-based and counseling activities [1,9,20,23,33]	When cultural, language, or confidentiality concerns affected patients’ ability to communicate or disclose [20,26] Patients with unstable mental health concerns (eg, suicidal ideation) [1] When physical examinations were necessary for screening tests or psychotherapy delivery [1]
	Post–test result follow-up	Predominately discussion tasks rather than physical examinations [22,23,26] When patients preferred to view test results via video compared with in person [26] Nonsensitive test results [9]	When discussing sensitive test results (eg, positive cancer diagnosis) [33] When explaining complex medical jargon used in test results [33]
	Postdischarge follow-up	When patients lived far away or had difficulty arranging a same-day visit or frequent follow-ups [23,26] Patients with pre-existing patient-provider relationships at the postoperative clinic [1]	When complex physical examinations were needed [18,23] When multiple care team members (eg, nurses) were needed to address physical aspects of care (eg, wound care) [23]
**Patient characteristics–based**			
	Existing patients (acute or existing concern)	Pre-existing patient-provider relationship [1,23,24,27] Established understanding of patients’ history [1,23,24,27] Pre-established rapport [1,23,24,27] Issues primarily reliant on assessing visual symptoms (eg, dermatological concerns) [32]	New diagnoses even with pre-existing patient-provider relationships [9] Severe concern that required more physical examinations (eg, chest pain or stomach pain) [30]
	New patients (acute or existing concern)	New patients when the consultation focused on pre-existing diagnosed concerns [1,23,25,27] Simple acute concerns (eg, dermatological concerns) that could be assessed using photos or video without complex physical examinations [1,23,25,27]	New diagnoses with no pre-existing patient-provider relationship or lack of knowledge of patient history [1,22] New patients with difficult or complex symptoms that relied on self-reported information or self-examinations [1,22] When patients were not forthcoming (eg, shyness or language or cultural barriers) [1,22] Technical issues affecting building rapport [33]

#### Chronic Condition Management

Chronic condition management visits in primary care were reported as being one of the visit types with the greatest number of studies reporting the use of telehealth during the COVID-19 pandemic (17/19, 89%). Of these 17 studies, 13 (76%) reported benefits and 6 (35%) reported drawbacks.

In the included studies, chronic condition management visits were often reported as suitable for telehealth because of *a pre-existing patient-provider relationship*, *established diagnosis*, *and lack of complex physical examinations.* Routine visits for chronic conditions (eg, diabetes checkups) could be facilitated using telehealth, as these tasks often relied on discussions (eg, diet and medication) [9,23,33]. Some of the examinations could be completed by patients at home under clinician guidance, such as foot examinations or weight measurements [1,18,20,22,27]. Patients could show their list of medications at home by reading the labels [22], and they could be educated on ways to use and administer medications at home (eg, asthma inhalers) and assisting with potential safety hazards or home support systems (eg, pets) [23]. Self-management education could also be enhanced if patients could share with their GPs during telehealth their home setting and at-home tools (eg, at-home blood pressure cuffs, glucose monitors, and heart rate monitors) [22].

A drawback of telehealth for chronic condition management in the included studies was when close monitoring (eg, complex comorbidities or cancer diagnoses) [30] or complex physical examinations (eg, pediatric examinations or smear examinations) were required [33]. Some patients reported being hesitant to use telehealth because of their unfamiliarity with the technology [21]. However, most patients with chronic conditions expressed high satisfaction and willingness to engage with telehealth again [31]. Patients with chronic conditions particularly favored the remote nature of telehealth as they were often at a higher risk of adverse symptoms if infected with COVID-19 when attending in-person clinics [21,23,28,29].

#### Existing Patients

Existing patient consultations were reported as being one of the visit types with the greatest number of studies reporting the use of telehealth during the COVID-19 pandemic (17/19, 89% of the included studies). Of these 17 studies, 11 (65%) reported benefits and 1 (6%) reported drawbacks.

In the included studies, telehealth was reported as suitable for visits with a pre-existing patient-provider relationship as clinicians understood the patients’ history and had a pre-established rapport [1,23,24,27,32]. Issues primarily reliant on assessing visual symptoms, such as dermatological concerns, could be shared with clinicians via photos or video [32]. In some cases, clinicians reported higher efficiency using telehealth. They could reduce the downtime involved in transiting between different patients during in-person encounters and see more patients via telehealth [9].

In the included studies, existing patients reported satisfaction with telehealth, especially for straightforward matters (eg, medication refill) and patients at high risk of COVID-19 [24,26,30]. However, a drawback of telehealth was when new diagnoses were involved, even among people with pre-existing patient-provider relationships, because of the poor ability to conduct physical examinations [9].

#### Medication Management

Medication management consultations were reported as being one of the visit types with the greatest number of studies reporting the use of telehealth during the COVID-19 pandemic (17/19, 89% of eligible studies). Of these 17 studies, 11 (65%) reported benefits and 3 (18%) reported drawbacks.

In the included studies, telehealth was reported as making medication reconciliations easier, improving patients’ adherence to their medications [1,9,22,32]. Telehealth was reported as supporting prescription refills for patients familiar with the medication’s side effects and risks and for straightforward health concerns such as oral contraceptives [9,31,32]. Patients reported being satisfied with their telehealth experience related to medications [1,18,25]. For example, patients could share their medications at home via video and image sharing with their clinicians. Furthermore, clinicians expressed greater relief when not being pressured to prescribe addictive drugs to at-risk patients during telehealth [22].

Drawbacks reported in the studies included concerns when physical examinations were necessary (eg, checking for infections when prescribing antibiotics) and prescription of new medications [1,24,30]. Poorer communication in patient education of medications was also observed in some telehealth consultations, potentially affecting patients’ understanding of their medications [1,24].

#### New Patients

New patient consultations with the use of telehealth during the COVID-19 pandemic were reported in 84% (16/19) of eligible studies. Of these 16 studies, 7 (44%) reported benefits and 9 (56%) reported drawbacks.

In the included studies, telehealth was reported as only suitable for new patients when the consultation focused on pre-existing diagnosed concerns, acute concerns (eg, dermatological concerns) that could be assessed via visual cues (such as via photo or video sharing), or when there was no need for physical examinations [1,23,25,27]. It is important to note that new patients are not always supported by health care reimbursement (eg, Medicare for Australian patients) outside certain criteria (ie, positive COVID-19 status, close contact, hot spot area, and emergency consultation), which affects the number of studies included for this visit type.

However, telehealth was reported as not suitable for new diagnoses when there was no pre-existing patient-provider relationship, lack of knowledge of patient history, or no pre-established patient rapport [1,22]. Telehealth for new patients would be particularly difficult when complex symptoms are involved or when patients are not forthcoming with their concerns (eg, feeling shy or experiencing language or cultural barriers). Managing new patients over telehealth would rely on trusting patients’ self-reported symptoms and patient-directed examination, which can become complicated when there is an absence of pre-existing knowledge of the patient [32]. In addition, technical problems within telehealth consultations can make building rapport with new patients even harder [33].

#### Mental Health and Behavioral Management

Mental health and behavioral management consultations with the use of telehealth during the COVID-19 pandemic were reported in 79% (15/19) of the included studies. Of these 15 studies, 11 (73%) reported benefits and 3 (20%) reported drawbacks.

In the included studies, telehealth was reported as only suitable for mental health and behavioral management when the consultation predominately focused on discussion and counseling activities [1,20,23,33]. Telehealth was suitable for patients with mild mental health issues (ie, patients not at risk to themselves or others), those without high cognitive impairments, and those who did not prefer a physical presence in consultations [20]. Studies involving patients with more complex mental health concerns referred to specialists (ie, psychiatrists) and participants in specialist mental health telehealth programs were excluded from this review. Patients with mental health concerns reported the benefits of reduced wait times when using telehealth during the COVID-19 pandemic, resulting in fewer barriers to accessing mental health care support [26,27]. Patients also reported being satisfied with their telehealth experience for mental health issues during the COVID-19 pandemic, particularly because of consultations being completed in a discreet manner (ie, the privacy of their own home) [1,9,24].

Drawbacks reported in the studies included patients’ hesitancy to disclose over telehealth because of the stigma around mental health concerns, cultural or language barriers, and confidentiality around disclosing sensitive matters where there was a lack of privacy at home [20,26]. It was challenging to conduct telehealth consultations with patients with unstable mental health concerns (eg, suicidal ideation) [1] or concerns requiring lengthier consultations [9]. There were mixed views about the need for physical examinations for screening tests [31].

#### Post–Test Result Follow-up

Post–test result follow-up consultations with the use of telehealth during the COVID-19 pandemic were reported in 74% (14/19) of the included studies. Of these 14 studies, 5 (36%) reported benefits and 3 (21%) reported drawbacks.

In the included studies, post–test result follow-up was often suitable for telehealth as the primary activity involved discussions of test results rather than conducting physical examinations [22,23,26,32,33]. For patients and clinicians, practices used procedures to ensure confidentiality via telehealth when receiving (and discussing) test results [23]. For example, some practices used confirmation ID numbers or asked patients to confirm their date of birth before revealing sensitive medical information because of the absence of in-person confirmation [23]. In some cases, telehealth also improved the ability to share test results with patients compared with in-person consultations (eg, screen sharing of test results with patients over video consultation) rather than the patient attempting to reach over to read the test result on the GP’s computer screen during in-person encounters [26].

A drawback of telehealth reported for this visit type was the poorer communication patterns observed when explaining to patients complex medical jargon used in test results. This is possibly related to the impersonal nature of telehealth, the inability to use visual aids, the lack of a physical presence, or other elements required to explain test results remotely [33]. Other clinic staff may communicate test results if results are satisfactory or do not require additional follow-up, resulting in minimal benefit and drawback findings reported for this visit type. Unsatisfactory results may lead to GP-patient consultations, possibly resulting in more drawbacks reported for this visit type.

#### Postdischarge Follow-up

Postdischarge follow-up consultations with the use of telehealth during the COVID-19 pandemic were reported in 37% (7/19) of the included studies. Of these 7 studies, 5 (71%) reported benefits and 3 (43%) reported drawbacks.

In the included studies, telehealth was reported as suitable for postdischarge follow-up visits when patients lived far away or had difficulty arranging same-day or frequent follow-up visits (eg, antenatal visits) [23,26]. Patients with pre-existing patient-provider relationships linked to the same postoperative clinic also reported satisfaction [1].

However, there was the drawback of it being harder to coordinate care [1]. This visit type often involved multiple care team members and complex physical examinations by various clinic members (eg, nurses and practitioners to address wound care) [18,23]. It was also challenging to share documentation from multiple team members [23].

### Benefits and Drawbacks of Using Telehealth in Primary Care During the COVID-19 Pandemic

This section outlines the benefits and drawbacks of using telehealth in primary care during the COVID-19 pandemic from patient and clinician perspectives reported according to the NQF Telehealth Framework. For more details on supporting evidence, please refer to Multimedia Appendix 9 [1,9,19,21-34].

#### Access to Care

The NQF outcome measure “Access to care” (ie, the ability to receive health services promptly and appropriately) was reported in 84% (16/19) of the included studies. A summary of benefits and drawbacks of telehealth per this outcome factor is provided in Table 5.

Both patients and clinicians reported the benefits of using telehealth to maintain timely and frequent contact, shortening wait times in between visits and having a satisfactory experience [1,22,25,26,30,32]. Patients particularly enjoyed the additional benefits of reduced travel time [32,33], the convenience of being at home [23,31], having quicker access to care for simple concerns [1,26,29], and being able to access care that was only available for a teleconsultation but not available for in-person consultations (eg, outside clinic opening hours) [19,30], whereas clinicians reported the benefits of seeing more patients using telehealth [28] and connecting with patients who preferred technology over in-person encounters [9,25].

**Table 5:** Benefits and drawbacks of telehealth according to the “Access to Care” outcome factor per perspective.

Perspective and benefits of telehealth		Drawbacks of telehealth
**Primary care clinician perspective**		
	Greater number of patients that can be seen using telehealth compared with in person (ie, teleconsultations tend to be shorter and more convenient, reducing cancelation rates) [25,26] Enables clinicians to connect with patients who may prefer technology over in-person encounters [25]	Harder to address language or cognition barriers [32] Need to address risks associated with digital platforms (eg, cyberattacks, security, and confidentiality in web-based communication) [25]
**Patient perspective**		
	Reduced travel time [31,34] Improved convenience [1,22,25,26,30,31] Ability to book consultations outside clinic hours [25,30] Ability to access care quicker owing to not requiring the same clinician for simple concerns [25,31,34]	Excludes and deters potentially at-risk patients who are not familiar with the technology [21,22]
**Both primary care clinician and patient perspective**		
	Satisfied with access and technical quality in most telehealth consultations [1,18] Timely and more frequent access to care for at-risk patients because of convenience and shortened wait times [1,26,27,30]	Insufficient technical support, infrastructure, or equipment to access telehealth [33] Varying complexity of telehealth systems needed because of different complexities in patients’ health conditions (eg, may require special equipment, hardware, or software or stronger internet access) [25]

However, patients and clinicians reported insufficient technical support, infrastructure, or equipment to access telehealth and difficulty with more complex telehealth systems that required special hardware or software support [25,32]. Some patients reported difficulty finding privacy at home to attend teleconsultations [21]. Some patients felt excluded or deterred from seeking help because of unfamiliarity with technology [21,22]. Some clinicians reported drawbacks of telehealth, such as it being harder to address language or cognition barriers with patients without physical cues [22] as well as feeling concerned with risks on digital platforms (eg, cyberattacks, security, and confidentiality in web-based communication) [25].

#### Effectiveness

The NQF outcome measure “Effectiveness” (ie, represents the systematic, clinical, operational, and technical success or barriers of telehealth) was reported in 84% (16/19) of the included studies. A summary of benefits and drawbacks of telehealth per this outcome factor is provided in Table 6. Both patients and clinicians reported that telehealth was suitable (ie, clinical appropriateness) for infections, dermatological concerns, renewal of prescriptions, or self-monitoring programs [25,32,33]. Most patients reported being sufficient at self-assessing whether they should seek a teleconsultation or an in-person consultation according to their health concerns [25]. Furthermore, patients could show their medication or self-care practices at home, allowing clinicians to better understand how their home environment may affect their self-management, thus improving clinicians’ advice dispensed to support their patients [1]. Clinicians also noted the benefits of sharing medical records with patients via screen sharing, improving their understanding [19].

However, telehealth was reported as not suitable for specific patient groups (eg, people with unstable mental concerns or low hearing and vision, young children unable to describe symptoms themselves, and people with cognitive impairment) [30] or for complex symptom presentations or diagnoses that required physical examinations (eg, chest pain, stomach pain, and potential new cancer) [1,9]. There is currently a lack of guidance on identifying and addressing severe adverse events that may occur because of telehealth (eg, lack of guidance on safety netting for teleconsultation, uncertainty about who else is also present but hiding during the teleconsultation, or recording consultations without consent) [1]. Furthermore, there is a tendency to rely more on patient-reported outcomes and patient-directed examinations during telehealth, affecting a GP’s assessment of the patient’s health status, which may inevitably result in in-person consultations later on despite having had a teleconsultation [25,33].

**Table 6:** Benefits and drawbacks of telehealth according to the “Effectiveness” outcome factor per perspective.

Perspective and benefits of telehealth			Drawbacks of telehealth
**Primary care clinician perspective**			
	Easier to share medical records with patients via screen sharing during video consultations [19] More efficient consultations with patients (ie, focused discussions and pretriaging procedures to preidentify concerns) [32]	Lack of guidance on appropriate ways to address serious adverse events related to telehealth [1] Increased reliance on trusting patients’ reported symptoms and self-examination assessment [33] Not suited for complex symptom presentations or diagnoses that require physical examinations (eg, chest pain, stomach pain, and potential new cancer) [1,9]	
**Patient perspective**			
	Improved ability for patients to self-manage their health because of their ability to share their medications or self-care practices at home with their clinicians [1] Most patients can self-assess the suitability of telehealth according to their health concerns [25]	Still requiring in-person consultations despite having had a teleconsultation already [25]	
**Both primary care clinician and patient perspective**			
	Perceived to be suitable for dermatological concerns and renewal of prescriptions or self-monitoring programs for improved patient outcomes [23,25]	Unsuitable for certain at-risk patient groups (eg, people who are mentally unstable or have low hearing and vision, young children, and people with cognitive impairment) [31,33]	

#### Experience

The NQF outcome measure “Experience” (ie, represents the usability and effect of telehealth on patients and providers) was reported in 89% (17/19) of the included studies. A summary of benefits and drawbacks of telehealth per this outcome factor is provided in Table 7.

Both clinicians and patients were satisfied with a perceived lower risk of infection transmission during the COVID-19 pandemic as a result of using telehealth [1,25-27,30,33]. Some reported feeling positive that they were able to maintain a patient-provider connection via telehealth during the COVID-19 pandemic [9,32,33]. Primary care clinicians reported several personal benefits of telehealth, including improved work-life balance and the ability to conduct some consultations more efficiently [33]. Clinicians also reported perceiving their patients as feeling more relaxed in their home environments compared with in-person consultations [33]. Overall, most patients reported having a satisfactory experience and a willingness to use telehealth again [21,24].

**Table 7:** Benefits and drawbacks of telehealth according to the “Experience” outcome factor per perspective.

Perspective and benefits of telehealth		Drawbacks of telehealth
**Primary care clinician perspective**		
	Improved work-life balance [33] Satisfied in perceiving their patients to be more relaxed in telehealth settings [32] Easier to conduct some consultations more efficiently [28]	Concerned about cultural and language barriers with patients [23] Lacking stimulating work for some clinicians as there is little in-person interaction with patients [9] Reliant on clinicians taking on multiple roles (eg, secretary, IT support, and clinician) [26,33]
**Patient perspective**		
	Satisfactory experience with telehealth consultations for surveyed patients [24,31] Surveyed patients willing to use telehealth again [21]	Lacking opportunity to develop in-person rapport because of cultural or language barriers, technological barriers, and confidentiality concerns [25,29] Lacking in establishing new patient-provider relationships [9,23] Impersonal in comparison with in-person care because of the remote nature of telehealth [27,31]
**Both primary care clinician and patient perspective**		
	Satisfied with lower risk of infection transmission [1,25-27,30] Positive patient-provider relationship for some patients as the personal connection was felt in teleconsultations [32]	Dissatisfied with the lack of in-person physical examinations [9,24,33]

As reported by both clinicians and patients, the main drawback of telehealth was dissatisfaction with a lack of in-person physical examinations [24]. Some clinicians faced the additional drawbacks of addressing language or cultural barriers without in-person cues [23] and the lack of stimulating work when there was little in-person interaction with patients [9]. In addition, telehealth sometimes required clinicians to take on multiple roles in the practice to ensure it ran smoothly (eg, secretary, IT support, and clinician) [26,33]. Patients similarly needed to combat barriers such as the lack of opportunity to develop rapport with their clinicians, impersonal consultations [27], language or cultural barriers to disclosing issues, technological barriers, and confidential concerns during web-based communication via telehealth [23].

#### Financial Impact or Cost

The NQF outcome measure “Financial Impact/Cost” (ie, potential cost savings or losses to patients, families, or providers) was reported in 63% (12/19) of the included studies. A summary of benefits and drawbacks of telehealth per this outcome factor is provided in Table 8. From the clinicians’ perspective, the infrastructure, processes, and long-term reimbursement models of telehealth were important considerations before its full potential and benefits could be unleashed [9,28]. For patients, removing the need to travel and reducing the loss of pay from taking time off work to attend in-person consultations were important drivers for choosing telehealth [34].

**Table 8:** Benefits and drawbacks of telehealth according to the “Financial Impact/Cost” outcome factor per perspective.

Perspective and benefits of telehealth			Drawbacks of telehealth
**Primary care clinician perspective**			
	Reduced telehealth setup costs because of existing infrastructure and processes (eg, adequate funding model and absence of billing or licensure restrictions) [9,18] Cost-effective in the long run because of reduced running costs compared with in-person consultations [9,22,31] Reimbursement model available for teleconsultations (eg, Medicare support in Australia) [28,29]	Expensive to set up a telehealth system from scratch [25] Long-term funding models are not globally determined, potentially opening up opportunities for commercial entities to exploit [25]	
**Patient perspective**			
	Some patients prefer telehealth consultations and are willing to pay [34] Some patients report that telehealth consultation fees are appropriate [24,31] Saving costs using telehealth (eg, travel costs to in-person clinics and for patients needing to take time off work for appointments) [34]	Mixed responses from some patients regarding willingness to pay for teleconsultation [26] Inappropriate telehealth consultation charges felt by some patients [24]	

However, issues relating to long-term models of financing and reimbursing telehealth remained a major concern [25]. For both patients and clinicians, there were concerns that remain to be researched about the expensive costs of acquiring the necessary software, hardware, and infrastructure to set up telehealth when it is unclear whether telehealth will remain a permanent service delivery mode in the long term. Furthermore, there is potential for commercial entities to exploit the charging or provision of telehealth when there remains uncertainty from the government on its long-term funding model [25]. There were mixed views regarding whether patients were willing to pay the same rate for telehealth consultations when compared with in-person consultations or an alternative appropriate cost [31].

## Discussion

### Principal Findings

To our knowledge, this is the first systematic review reporting visit types in primary care where telehealth was used during the COVID-19 pandemic. Most of the included studies (13/19, 68%) were level-4 evidence (cohort studies, interviews, and surveys), reflecting the early experience of the pandemic. Seven primary care visit types were identified: chronic condition management (17/19, 89%), existing patients (17/19, 89%), medication management (17/19, 89%), new patients (16/19, 84%), mental health and behavioral management (15/19, 79%), post–test result follow-up (14/19, 74%), and postdischarge follow-up (7/19, 37%). Benefits and drawbacks were reported across all visit types, with chronic condition management visits being one of the visit types with use of telehealth reporting the greatest number of studies during the pandemic (17/19, 89%). Reasons for why telehealth was deemed suitable for chronic condition management visits included patients having pre-existing diagnoses, established patient-provider relationships, and lack of complex physical examinations required. Insights into both the primary care clinician and patient perspective of telehealth use for specific visit types (ie, access to care, effectiveness, experience, and financial impact or cost) were also provided. Overall, benefits of telehealth included improved convenience, focused discussions, and continuity of care despite social distancing practices during the COVID-19 pandemic. Drawbacks of telehealth included technical barriers, impersonal interactions, and semi-established reimbursement models.

### Strengths and Limitations

The strengths of this study include following a rigorous approach at all stages of the systematic review. For example, a wide range of academic databases and gray literature were searched to ensure great coverage of literature. In total, 3 independent researchers following predetermined eligibility criteria were involved in article screening to reduce the risks of selection bias. Data extraction templates (eg, the Joanna Briggs Institute data abstraction form) were used to standardize reporting of findings between studies. Well-established tools (eg, GRADE and the Mixed Methods Appraisal Tool) were used to conduct a critical appraisal and assess levels of evidence for each included study. Furthermore, definitions and terminologies from widely accepted frameworks in the telehealth and primary care communities (such as the NQF Telehealth Framework, the RACGP, and the Medicare Benefits Schedule) were used to ease the translation of our review.

The limitations of this review include restricting it to studies between late 2019 and August 2022 as definitions of the COVID-19 era, limiting it to studies written in English, and the decision to focus on broadness rather than narrowness in our search strategy. Publication bias (ie, the tendency to report positive results) may be present in the included studies because of the novel adoption of telehealth during the COVID-19 pandemic and growing interest in this research space [44]. Since our review, additional studies may have been published focusing on the experience of telehealth as GPs and patients have become more experienced with its use within routine primary care settings. Thus, despite multiple search cycles, our review may only reflect early experiences of telehealth during the COVID-19 era. In addition, this systematic review focuses on the early experience of the COVID-19 pandemic, where primary studies on clinical outcomes of using telehealth during the pandemic were not yet available. Future reviews should examine the long-term clinical outcomes of patients using telehealth (or hybrid models of care) in primary care settings. Our search strategy did not use keywords related to specific visit types in primary care. Instead, we chose to focus broadly on primary care to ensure we captured all studies with telehealth support conducted in primary care during the pandemic that were reported. Future reviews could include non-English studies or specific visit types to increase the generalizability and scope of the findings.

### Comparison With Prior Work

Before the COVID-19 pandemic, studies on telehealth focused on issues such as particular visit types (eg, medication reviews or chronic condition management visits) [45,49], patient satisfaction [46,47], or nonsynchronous patient-provider communication (eg, e-consultation portals) [48]. For example, a review by Polisina et al [45] explored the use of an at-home management program for a chronic condition such as diabetes. A systematic review by Hanjani et al [49] focused explicitly on medication reviews via telehealth and identified similar facilitators and barriers to those of our review. Most of the benefits and drawbacks of telehealth reported in this review, such as ease of use, reduced travel times, low cost, and improved communication (in some instances), were also found by Kruse et al [46] in their systematic review. Other studies such as that by Hollander and Goldwater [47] examined the use of telehealth in orthopedic surgery, and Villarreal et al [48] reviewed mobile systems designed for health care monitoring.

Our systematic review focused on studies published in the COVID-19 era to consider how telehealth was used in primary care during the pandemic. A recent systematic review by Snoswell et al [15] aligns with our recommendations, stating that telehealth services are equivalent to or (at times) more effective than in-person care. However, Snoswell et al [15] did not report telehealth experience during the COVID-19 pandemic, instead focusing on studies from 2010 to 2019. A recent systematic review from Carrillo de Albornoz et al [50] evaluated the effectiveness of teleconsultations in primary care and mental health services in comparison with in-person visits, providing similar insights into the usability of telehealth as an effective alternative to in-person consultations. However, although this study was published following the emergence of COVID-19, the included studies were not conducted during the COVID-19 pandemic and, therefore, this study does not reflect on the effectiveness of teleconsultations in light of the pandemic.

A rapid scoping review by Jonnagaddala et al [51] explored facilitators and inhibitors of primary care informatics to COVID-19 in Australia. Similarly, we found limited high-quality evidence on the effectiveness, access, equity, utility, safety, and quality of digital health during the COVID-19 pandemic. However, our review differs in the systematic review approach. We identified 7 visit types where telehealth was used in primary care during the pandemic, outlining the benefits and drawbacks of using telehealth for each visit type and in primary care overall.

### Implications for Digital Health, Clinical Practice, and Future Research

In total, 3 key insights have emerged from this review.

#### Key Insight 1: Rigorous Research Is Needed to Investigate Which Visit Types Are Indeed Suitable for Telehealth in Primary care

The results of our systematic review identified a lack of quality evidence on primary care visit types suitable for telehealth. Most of the included studies (13/19, 68%) were level-4 evidence (ie, case series or cross-sectional studies), which are subject to self-report bias. Furthermore, there is a lack of focus on how telehealth was used for different visit types in primary care. The saturation of level-4 evidence in this space conveys that these study designs are indeed the current state of the art, presumably from the relatively short time since the start of the pandemic as well as the lack of ability to conduct follow-up or person-facing studies because of social distancing restrictions. As we move into the era of living with COVID-19, studies with a longitudinal follow-up that focus on specific visit types are required to assess the long-term suitability of telehealth in primary care. In addition, there was a lack of research in the included studies reporting clinical outcomes related to telehealth use during the pandemic, presumably because, at the time of searching and writing (ie, early phases of the COVID-19 pandemic), studies assessing clinical outcomes of using telehealth during the pandemic would not have yet been available. When those studies become available, future systematic reviews may wish to assess clinical outcomes of using telehealth during the pandemic so that the findings in this review reporting the suitability of telehealth for primary care visits can be validated.

#### Key Insight 2: Long-term Models of Telehealth and Their Impact on Patient Outcomes and Health Service Use

As a result of COVID-19, several countries (eg, Australia, the United States, and the United Kingdom) have introduced permanent or long-term funding for telehealth in primary care. For example, the Australian government introduced long-term funding for telehealth in December 2021 to align with initiatives to reduce community COVID-19 transmission [52]. Australians have welcomed telehealth consultations, with >86 million primary care telehealth consultations completed in Australia since the beginning of the COVID-19 pandemic [8]. Other countries such as the United States and the United Kingdom are exploring a permanent funding scheme for telehealth within their existing health care models. Almost every state Medicaid program has a reimbursement coverage account for telehealth services in the United States [53]. The Centers for Disease Control and Prevention also introduced multiple waivers during the COVID-19 pandemic to grant payment parity for telehealth [54].

Before the pandemic, telehealth policies in the United Kingdom alone were underdetermined across England, Wales, Northern Ireland, and Scotland. Challenges related to outdated systems and underinvestment in telehealth have hindered the progress of digitization [55]. During the COVID-19 pandemic, health care services under the National Health Service took a “total triage” approach where all patients were referred first to telehealth services over face-to-face services [55]. According to the Health Foundation, this initiative has caused a rapid and significant increase in telehealth use, reporting the highest-ever number of telephone consultations in English primary care as a consequence of the pandemic [56]. For example, a videoconferencing telehealth platform called “Near Me” reported having been used by approximately 300 people per week at the start of 2020, rising to approximately 20,000 appointments every week by mid-2020 [55]. By July 2021, >1 million appointments were delivered via telehealth services [55]. Furthermore, 11.4 million telephone consultations were reported to have been completed in March 2021 compared with 3.5 million in March 2019 [56]. This rapid and unforeseen uptake of telehealth services raises questions as to whether unintended consequences and safety risks may have been introduced as well [55].

Governments have recognized the value of telehealth during the pandemic, especially for patients who struggle with mobility [1,25], live remotely or rurally [15], or are unable to find suitable times to attend in-person consultations [8], regardless of their COVID-19 status. Future research ought to examine how long-term funding models of telehealth affect patient outcomes, help-seeking behaviors, and health service use patterns. For example, further research is required to compare the health outcomes and quality of care between patients who primarily use telehealth experiences versus those who use in-person care. In addition, further research is required to analyze how changes in health service use patterns because of routine telehealth use affect the funneling of resources, particularly training opportunities for health care providers on how to use telehealth optimally and the communication skills required in telehealth.

Furthermore, there is the additional consideration of how designs of telehealth need to evolve with emerging safety, ethical, and equitable concerns, for example, how to ensure that all patients can equally access care, regardless of the digital divide, if more resources are directed to providing telehealth over in-person services. In addition, further research is required to explore how to support patient-provider relationships when care is delivered across a blended model of approaches, as well as further research into appropriate safety-netting practices during teleconsultations [57].

#### Key Insight 3: Patient Safety at Home Is Paramount as Care and Technology Are Increasingly Used Outside Clinical Settings

Increasingly, care is moved closer to patients’ homes, blended with technology. The pandemic has accelerated the movement of blending care and technology at home. For example, home oximetry monitoring programs have been introduced for monitoring positive COVID-19 patients in the United Kingdom, and a recent prospective study has reported patient satisfaction and early success [58]. Other digital health services have also been increasingly introduced for use outside clinical settings, such as assistive technology to support independent living at home [13,59], remote monitoring mobile apps [60,61], and e–mental health services (eg, Betterhelp and Headspace) [52].

Introducing technologies directly into patients’ homes as part of routine service delivery may encourage more frequent monitoring of signs and symptoms. However, patient-facing medical devices and at-home care can introduce a new dimension of patient risk [62]. Historically, the role of conducting physical examinations and use of medical devices was reserved for health care professionals [63]. However, with telehealth and remote care services, the responsibility of physical examination and monitoring falls onto the patient, requiring patients to have the necessary knowledge and skills to conduct these previously clinician-directed tasks effectively by themselves or be aware of when to seek additional assistance [63]. As a result, patients could become vulnerable to unanticipated risks such as inaccurate self-examination [64,65], unreliable patient self-reports, reduced person-centered care because of language or cognitive barriers, inability to conduct a physical examination properly, or incapacity to receive care properly because of technological limitations [62,63]. Further investigation is required to identify the types of adverse events that can occur during remote care (eg, whether people are using technologies as intended or whether technologies are introducing unintended consequences) and ways to combat these adverse events [64].

### Conclusions

This systematic review identified 7 visit types in primary care with telehealth support during the COVID-19 pandemic, with the greatest number of studies reporting benefit findings for *chronic condition management* visits (17/19, 89%). Benefits and drawbacks of using telehealth were reported across different visit types from patient and clinician perspectives, as well as the circumstances in which telehealth was found to be suitable (or not) for each primary care visit type. As telehealth potentially becomes a long-term care delivery model, improving telehealth consultation delivery while monitoring patient safety at home will emerge as an important priority area.

## Appendix

Multimedia Appendix 1 PRISMA (Preferred Reporting Items for Systematic Reviews and Meta-Analyses) checklist.

Multimedia Appendix 2 Study Characteristics.

Multimedia Appendix 3 Search Strategy.

Multimedia Appendix 4 Eligibility Criteria.

Multimedia Appendix 5 Data Extraction Template.

Multimedia Appendix 6 Risk and Bias Critical Appraisal.

Multimedia Appendix 7 Visit Type Medicare Descriptions.

Multimedia Appendix 8 Supporting Evidence of Suitability of Telehealth for each Visit Type during COVID-19.

Multimedia Appendix 9 Supporting Evidence of Benefit and Drawback of using Telehealth in Primary care during COVID-19 according to outcomes of the NQF Framework.

## Abbreviations

GP: general practitioner
GRADE: Grading of Recommendations Assessment, Development, and Evaluation
NQF: National Quality Forum
PRISMA: Preferred Reporting Items for Systematic Reviews and Meta-Analyses
RACGP: Royal Australian College of General Practitioners
