# Supplementary material for: Visit Types in Primary Care With Telehealth Use During the COVID-19 Pandemic: Systematic Review
Source: JMIR Med Inform. 2022 Nov 28;10(11):e40469. doi: 10.2196/40469 (PMC9745650; doi:10.2196/40469)
Supplement: Multimedia Appendix 2 [file medinform_v10i11e40469_app2.docx]

# Appendix 2. Study Characteristics

## Table 2A. Study Characteristics of Included Studies (n = 19)

| **Source (Year, Country)** | **Study Design** | **Study Population** | **Health Conditions Featured Within Study** | **Visit Type Featured Within Study** | | **Telemedicine Approach Used** | **NQF Framework Outcome Measures*** | **Clinical Measures** |
| --- | --- | --- | --- | --- | --- | --- | --- | --- |
|  |  |  |  | **Patient Type** | **Task** |  |  |  |
| **Jetty et al. (2021, United States of America)** | Cross- Sectional Survey | - Primary care physicians (n = 677) - Patient-physician encounters (n = 330, 605, 000) | - Cancer - Cardiovascular - Digestive - Endocrine/Metabolic and Nutritional - Eye - Female Genital - General & Unspecified - Male Genital - Musculoskeletal - Neurological - Pregnancy, Childbearing & Family Planning - Psychological - Respiratory - Skin - Urological | - Existing Patient (Acute or Existing Problem/Concern) - New Patient (Acute or Existing Problem/Concern) | - Chronic Condition Management - Medication Management - Mental Health/Behavioural Management - Post-Surgery/Hospital Discharge - Post-Test Results | - Telephone - E-mail messaging | - *Access to care*: Use of technology - *Effectiveness*: Perception of proportions of amendable services to telehealth - *Financial Impact/Cost*: Reimbursement types | - N/A |
| **Murphy et al. (2021, United Kingdom)** | Mixed Methods Study | - Primary care worker (n= 87) - Based on 218 GP consultations | - Cancer - Cardiovascular - Endocrine/Metabolic and Nutritional - General & Unspecified - Musculoskeletal - Psychological - Respiratory - Urological | - Not Reported | - Chronic Condition Management - Medication Management - Mental Health/Behavioural Management - Post-Test Results - Post- Surgery/Hospital Discharge | - Email messaging - Telephone - Text messaging - Video | - *Effectiveness*: perception of suitability of telehealth, perception of safety by primary care workers - *Experience*: Primary care worker preference for telehealth and willingness to use telehealth - *Financial Impact/Cost*: Willingness to pay for telehealth | - Clinical status (mental health status, shielding status) |
| **Johnsen et al. (2021, Norway)** | Cross Sectional Survey | General practitioners (n = 1237) | - Cancer - Cardiovascular - Digestive - Ear - Endocrine/Metabolic and Nutritional - Eye - Female Genital - General & Unspecified - Male Genital - Musculoskeletal - Neurological - Pregnancy, Childbearing & Family Planning - Psychological - Urological | - Existing Patient (Acute or Existing Problem/Concern) - New Patient (Acute or Existing Problem/Concern) | - Chronic Condition Management - Medication Management - Mental Health/Behavioural Management - Post-Surgery/Hospital Discharge - Post-Test Results | - Video | - *Access to care*: Use of technology, reason for contact, reason for contact to telehealth - *Effectiveness*: perception of severity of health concerns suitable for telehealth, perception of concerns of not picking up serious illness during tele-consultations, perceived safety of telehealth from GPs and patients - *Experience*:   GP satisfaction with technology, perception of patient satisfaction, perception of impact on patient-clinician relationships | - N/A |
| **Grossman et al. (2020, Israel)** | Cross Sectional Survey | Primary care paediatrician (n = 169) | - Ear - Respiratory | - Existing Patient (Acute or Existing Problem/Concern) - New Patient (Acute or Existing Problem/Concern) | Not reported | - Telephone - Text messaging - Video | - *Access to care*: Use of technology, total visits, timeliness of care, reason for contact - *Effectiveness*: Patterns of clinical decision making, perception of severity of health concerns suitable for telehealth, perception of concerns of not picking up serious illness during tele-consultations | - Requesting for x-ray score (yes/no)_ - Prescription of antibiotics score (yes/no) |
| **Imlach et al. (2020, New Zealand)** | Mixed-methods Study (Cross-sectional survey, in-depth interviews qualitative analysis) | Primary care patients (n = 1010 survey respondents, 38 interviews) | - Cancer - Cardiovascular - Endocrine/Metabolic and Nutritional - General & Unspecified - Musculoskeletal - Psychological - Respiratory | - Existing Patient (Acute or Existing Problem/Concern) - New Patient (Acute or Existing Problem/Concern) | - Chronic Condition Management - Medication Management - Mental Health/Behavioural Management - Post-Surgery/Hospital Discharge - Post-Test Results | - Email - Telephone - Video | - *Access to care*: Convenience, use of technology, technological barriers, timeliness of care, reasons for contact - *Effectiveness*: Perception of safety from patients - *Experience*: Patient preference for and willingness to use telehealth , perception of impact on patient-clinician relationships, patient preferences of care - *Financial Impact/Cost*: Ability to pay for sessions, views on value of telehealth | - N/A |
| **Gomez et al. (2021, United States of America)** | Cross Sectional semi-structured interviews | Primary care physicians (n =15) | - Cardiovascular - Endocrine/Metabolic and Nutritional - General & Unspecified - Respiratory - Psychological | - Existing Patient (Acute or Existing Problem/Concern) - New Patient (Acute or Existing Problem/Concern) | - Chronic Condition Management - Medication Management - Mental Health/Behavioural Management - Post-Test Results | - Telephone - Video | - *Access to care:* Perception of patient access to care, convenience, timeliness of care - *Effectiveness*: Perceived suitability of telehealth from patient perspective, patterns of clinical decision making, perception of appropriateness of diagnosis and treatment made via telehealth, perception of safety from primary care clinician - *Experience*: Primary care clinicians experience - *Financial Impact/Cost*: Reimbursement types | - N/A |
| **Hasani et al. (2020, Oman)** | Cross Sectional semi-structured interviews | Primary Care workers (n = 22, 5 were GPs) | - General & Unspecified | - Existing Patient (Acute or Existing Problem/Concern) - New Patient (Acute or Existing Problem/Concern) | - Chronic Condition Management - Medication Management - Mental Health/Behavioural Management - Post-Surgery/Hospital Discharge - Post-Test Results | - Telephone | - *Access to care*: Technical support available to primary care clinicians - *Experience*: Perception of measures for privacy and confidentiality, experience with clinical infrastructure and guidelines - *Financial Impact/Cost*: Financial support available to primary care clinicians and patients | - N/A |
| **Jabbarpour et al. (2021, United States of America)** | Cross Sectional Survey | Primary Care Worker, patient-physician encounters (n = 850 million) | - Cancer - Cardiovascular - Digestive - Eye - Endocrine/Metabolic and Nutritional - Female Genital - General & Unspecified - Male Genital - Musculoskeletal - Neurological - Pregnancy, Childbearing & Family Planning - Psychological - Respiratory - Skin - Urological | - Existing Patient (Acute or Existing Problem/Concern) - New Patient (Acute or Existing Problem/Concern) | - Chronic Condition Management - Medication Management - Mental Health/Behavioural Management - Post-Test Results | - Email - Telephone - Text messaging - Video | - *Effectiveness*: Perceived suitability of telehealth from patient perspective | - Number of wellness visits and immunisations - Number of examinations, procedures, treatments, and lab tests |
| **Schwelberger et al. (2020, United States of America)** | Retrospective analysis | Primary care clinician practices (n = 45) | - Digestive - Ear - Eye - General & Unspecified - Pregnancy, Childbearing & Family Planning - Psychological - Respiratory - Skin - Urological | - Existing Patient (Acute or Existing Problem/Concern) - New Patient (Acute or Existing Problem/Concern) | - Chronic Condition Management - Medication Management - Mental Health/Behavioural Management - Post-Test Results | - Email messaging - Telephone - Text messaging - Video | - *Access to care*: Technology useability questionnaire, number of telemedicine visits, timeliness of care, reasons for contact - *Effectiveness*: Perception of telehealth usability and usefulness, perceived suitability of telehealth from patient perspective, perception of safety through electronic health record data analysis - *Experience*: Telemedicine satisfaction (measured via questionnaire) - *Financial Impact/Cost*: Insurance type and insurance cost | - N/A |
| **Van de Poll-Franse et al. (2021, Netherlands)** | Cross Sectional Survey | Primary care patients (n = 4094) | - Cancer - General & Unspecified | - Existing Patient (Acute or Existing Problem/Concern) - New Patient (Acute or Existing Problem/Concern) | - Chronic Condition Management - Medication Management - Post-Test Results | - Telephone - Video | - *Access to care*: Use of technology, reasons for contact - *Effectiveness*: Perceived suitability of telehealth from patient perspective - *Experience*: Perception of patient privacy, patient preference | - Differences in scores on quality of life, anxiety/depression, and loneliness between patients and age-matched & sex-matched participants |
| **RACGP (2020, Australia)** | Clinical Practice Guidelines | No study population | - Cancer - Cardiovascular - Digestive - Ear - Eye - Endocrine/Metabolic and Nutritional - Female Genital - General & Unspecified - Male Genital - Musculoskeletal - Neurological - Pregnancy, Childbearing & Family Planning - Psychological - Respiratory - Skin - Urological | - Existing Patient (Acute or Existing Problem/Concern) - New Patient (Acute or Existing Problem/Concern) | - Chronic Condition Management - Medication Management - Mental Health/Behavioural - Post-Surgery/Hospital Discharge - Post-Test Results | - Telephone - Video | - *Access to care*: Use of technology, reasons for contact - *Financial Impact/Cost*: Reimbursement types and reimbursement cost | - N/A |
| **Department of Health, MBS (2020, Australia)** | Clinical Practice Guidelines | No study population | - Cardiovascular - Digestive - Ear - Endocrine/Metabolic and Nutritional - Female Genital - General & Unspecified - Male Genital - Musculoskeletal - Neurological - Pregnancy, Childbearing & Family Planning - Psychological - Respiratory - Skin - Urological | - Existing Patient (Acute or Existing Problem/Concern) - New Patient (Acute or Existing Problem/Concern) | - Chronic Condition Management - Medication Management - Mental Health/Behavioural - Post-Surgery/Hospital Discharge - Post-Test Results | - Telephone - Video | - *Financial Impact/Cost*: Reimbursement types and reimbursement cost | - N/A |
| **Gabrielsson-Jarhult, Kjellstrom, and Josefsson (2021, Sweden)** | Mixed methods study  Qualitative interviews and quantitative registry study of data | - Patients (n=26) - Quantitative registry study of data users (n=10,400) | - Digestive - Endocrine/Metabolic and Nutritional - General & Unspecified - Pregnancy, Childbearing & Family Planning - Skin | - New Patient (Acute or Existing Problem/Concern) | - Chronic Condition Management - Medication Management | - Telephone - Text messaging | - *Access to care*: Perception of accessibility to care, convenience, number of telemedicine visits, timeliness of care - *Effectiveness*: Perception of impact to care quality, analysis of care patterns in telehealth versus in-person - *Experience*: Users and professionals experience with telehealth, perception of user satisfaction - *Financial impact/cost*: Perception of value of telehealth | - N/A |
| **Javanparast et al. 2021 (Australia)** | Semi-structured telephone interviews | Patients (n=9) | - Cancer - Cardiovascular - Digestive - Endocrine/Metabolic and Nutritional - General & Unspecified - Musculoskeletal - Neurological - Psychological - Respiratory - Skin - Urological | - Existing Patient (Acute or Existing Concern) | - Chronic condition Management - Medication Management - Mental Health/Behavioural Concern | - Telephone | - *Access to Care*: Perceived access to care, number of visits, reasons for contact - *Effectiveness*: Perception of safety (use of safety measures), perceived suitability of telehealth from patient perspective - *Experience*: Patient experience with telehealth services, perception of impact on patient-provider relationship - *Financial Impact/Cost*: Use of Reimbursement and bulkbilling availability | - N/A |
| **Mozes et al. 2022 (Israel)** | Mixed methods study  Discrete choice experiment.  Literature review, focus group interviews, attribute selection. | - Patients (n=26) - Physicians (n=33) | - Cancer - Cardiovascular - Endocrine/Metabolic and Nutritional - General & Unspecified - Musculoskeletal - Respiratory - Skin | - Existing Patient (Acute or Existing Concern) | - Chronic condition Management - Medication Management | - Email messaging - Telephone - Text messaging - Video | - *Access to Care*: In-person follow up visits post telehealth consultations, timeliness of care - *Effectiveness*: Perception of suitability of telehealth per the severity of patient concerns, risk of infection comparison between in-person and telehealth - *Experience*: perception of impact on patient-clinician relationships | - N/A |
| **De Guzman et al. 2022 (Australia)** | Qualitative study  Semi-structured interviews | - General Practitioners (n=14) | - Cancer - Cardiovascular - Digestive - Endocrine/Metabolic and Nutritional - General & Unspecified - Musculoskeletal - Neurological - Pregnancy, Childbearing & Family Planning - Psychological - Respiratory - Skin | - Existing Patient (Acute or Existing Concern) - New Patient (Acute or Existing Concern) | - Chronic condition Management - Medication Management - Mental health/behavioural management - Post-test results | - Telephone - Video | - *Access to Care*: Use of technology - *Effectiveness*: Provision of quality care, perception of safety from GPs - *Experience*: Experience of telehealth from GP, perception of patient experiences and preferences - *Financial Impact/Cost*: GP reported financial pressures, out of pocket clinic costs, reimbursement availability | - N/A |
| **Due et al. 2021 (Denmark)** | Qualitative study  Interviews | - General practitioners (n=13) | - Cancer - Cardiovascular - Endocrine/Metabolic and Nutritional - General & Unspecified - Musculoskeletal - Psychological - Respiratory - Skin | - New Patient (Acute or Existing Concern) | - Chronic condition Management - Medication management - Mental health/behavioural management - Post-test results | - Telephone - Video | - *Access to Care*: Technical limitations, timeliness of care, reason for contact - *Effectiveness*: Perception of safety measures (covid safety measures, use of PPE) from GP, perception of appropriate reasoning for telehealth consultations, relational and nonverbal limitations of telehealth - *Experience*: Perception of patient experience, GP experience, experiences of clinical quality/ challenges/preferences | - Clinical examinations and treatments provided |
| **Assing Hvidt et al. 2021 (Denmark)** | Qualitative study  Semi-structured interviews | - Patients (n=27) | - Cancer - 1Endocrine/Metabolic and Nutritional - General & Unspecified - Psychological - Respiratory - Skin - Urological | - Existing Patient (Acute or Existing Concern) - New Patient (Acute or Existing Concern) | - Chronic condition Management - Medication Management - Mental health/behavioural Management - Post-test results | - Video | - *Access to Care*: Reasons for contact, convenience, flexibility, use of technology, timeliness of care, number of telemedicine visits - *Effectiveness*: Use and perceived quality of communication from patients view, perception of improvement of digital technology involved in telehealth, perception of the efficiency of care - *Experience*: Patient experience, perception of impact on patient-clinician relationships, | - N/A |
| **Manksi-Nankervis et al. 2021 (Australia)** | Cross-sectional survey study | - Patients (n=499) | - Cancer - Cardiovascular - Endocrine/Metabolic and Nutritional - General & Unspecified - Musculoskeletal - Psychological - Respiratory - Skin | - Existing Patient (Acute or Existing Concern) - New Patient (Acute or Existing Concern) | - Mental health/behavioural management | - Video | - *Access to Care*: Travel time, convenience, timeliness of care, reasons for contact - *Effectiveness*: Perception of safety and privacy from patients - *Experience*: Consumer experience and preferences, perception of impact on patient-clinician relationships, acceptability of telehealth - *Financial Impact/Cost*: Estimated savings and cost analysis | - N/A |

Note: Table inputs for respective columns are in alphabetical order. Sources listed in no specific order.

*For the NQF outcome measure *‘effectiveness’* this study characteristics table does not include the clinical measures/outcomes reported per the NQF outcome measure outlined definition. For this table only, clinical outcomes/measures will be listed in the ‘*clinical measures’* column of this table.
