# Supplementary material for: Visit Types in Primary Care With Telehealth Use During the COVID-19 Pandemic: Systematic Review
Source: JMIR Med Inform. 2022 Nov 28;10(11):e40469. doi: 10.2196/40469 (PMC9745650; doi:10.2196/40469)
Supplement: Multimedia Appendix 3 [file medinform_v10i11e40469_app3.docx]

# Appendix 3. Search Strategy

## Table 3A. Search Strategy Template for Searchable Terms Breakdown


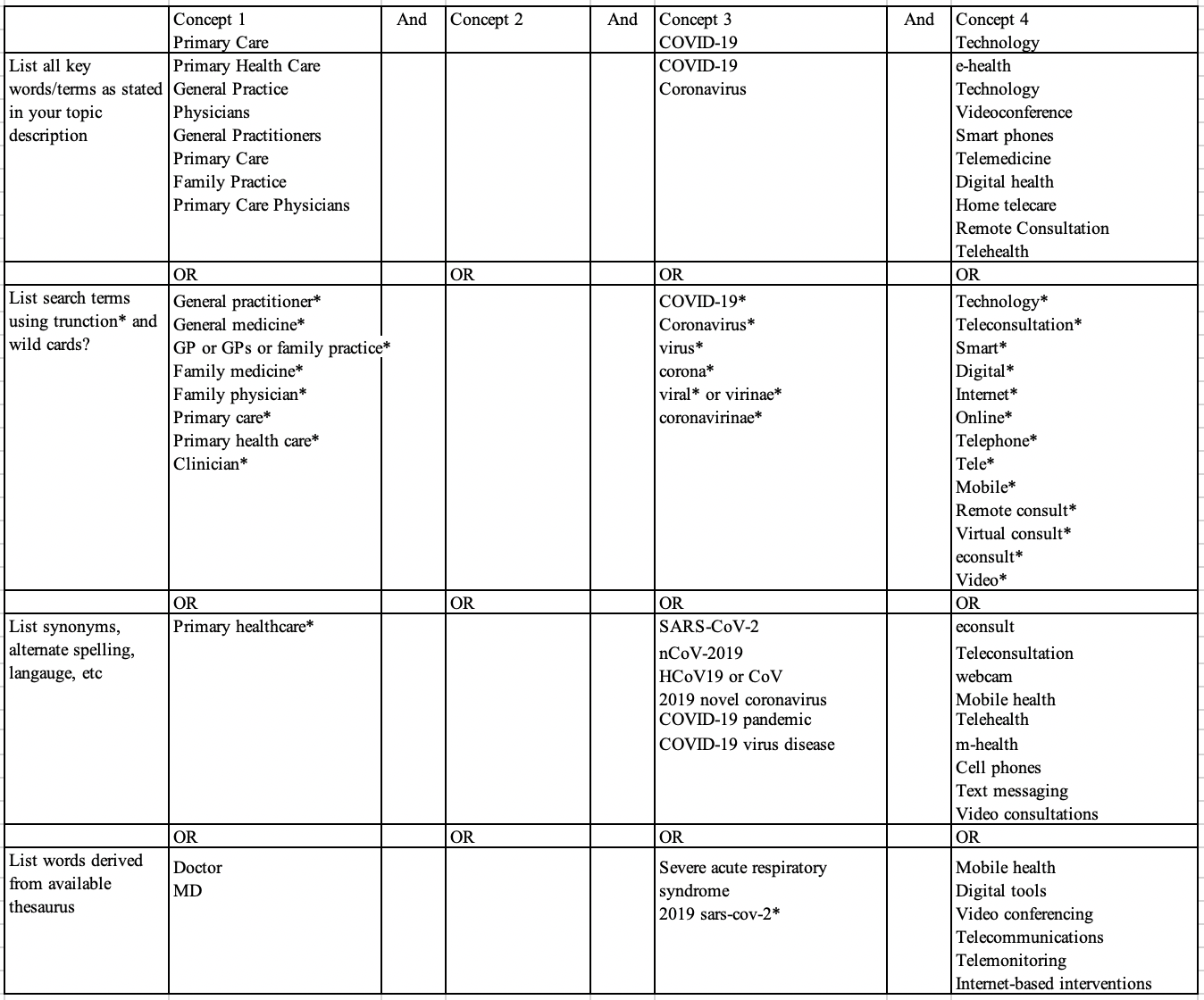


## Table 3B. Search Strategy for Ovid (MEDLINE) database

| 1 | Telemedicine/ or Internet/ or technology/ or digital technology/ or Videoconferencing/ or Remote Consultation/ or Online Systems/ |
| --- | --- |
| 2 | (e-health or videoconference or health telecare or digital health or online or mobile or telephone or e-consult* or teleconsultation or video or suitability or visit).mp. |
| 3 | Text Messaging/ or Mobile applications/ |
| 4 | General Practice/ or General Practitioners/ or Family Practice/ or Primary Health Care/ or Physicians, Family/ |
| 5 | (General practitioner or family practice or primary health care or family medicine or general medicine).mp. |
| 6 | 2 OR 3 |
| 7 | 1 AND 6 |
| 8 | 4 OR 5 |
| 9 | 7 AND 8 |
| 10 | Limit 9 to (English language and yr= “2020=Current”) |

## Table 3C. Search Strategy for CINALH Complete Database

| S1 | (MH "Telehealth+") OR (MH "Telemedicine+") OR "telemedicine or telehealth or telecare" OR "teleconsultation" OR (MH "Remote Consultation") | **Expanders** - Apply equivalent subjects  **Search modes** - Boolean/Phrase |
| --- | --- | --- |
| S2 | (MH "Internet+") OR "internet" OR (MH "Internet-Based Intervention") | **Expanders** - Apply equivalent subjects  **Search modes** - Boolean/Phrase |
| S3 | "videoconferencing" OR "videotelephony" OR "video conference" OR "telephone"  OR “suitability” or “visit” | **Expanders** - Apply equivalent subjects  **Search modes** - Boolean/Phrase |
| S4 | (MH "Remote Consultation") OR "remote consultation" | **Expanders** - Apply equivalent subjects  **Search modes** - Boolean/Phrase |
| S5 | "e-health or ehealth or digital health or telemedicine or telehealth" | **Expanders** - Apply equivalent subjects  **Search modes** - Boolean/Phrase |
| S6 | "general practice or gp or primary care or primary healthcare or primary health care" OR (MH "Primary Health Care") | **Expanders** - Apply equivalent subjects  **Search modes** - Boolean/Phrase |
| S7 | (MH "Physicians, Family") | **Expanders** - Apply equivalent subjects  **Search modes** - Boolean/Phrase |
| S8 | S2 OR S3 OR S4 OR S5 | **Expanders** - Apply equivalent subjects  **Search modes** - Boolean/Phrase |
| S9 | S1 AND S8 | **Expanders** - Apply equivalent subjects  **Search modes** - Boolean/Phrase |
| S10 | S6 OR S7 | **Expanders** - Apply equivalent subjects  **Search modes** - Boolean/Phrase |
| S11 | S9 AND S10 | **Expanders** - Apply equivalent subjects  **Search modes** - Boolean/Phrase |
| S12 | S9 AND S10 | **Limiters** - Published Date: 20190101-20211231  **Expanders** - Apply equivalent subjects  **Search modes** - Boolean/Phrase  Revised search in August 2022 searched with Published Date: 20191201-20220808 |
| S13 | S9 AND S10 | **Limiters** - Published Date: 20190101-20211231  **Expanders** - Apply equivalent subjects  **Narrow by Language:**- english  **Search modes** - Boolean/Phrase  Revised search in August 2022 searched with Published Date: 20191201-20220808 |

*Search Strategy for ProQuest*

(mesh.Exact("Telemedicine" OR "Telephone" OR "Videoconferencing" OR "Internet" OR "Remote Consultation")) AND ("Tele*" OR "e-consult" OR "e-consultation" OR "e-consulting" OR "video consult" OR "telehealth" OR "Telemedicine" Or “suitability” OR “visit”) AND (mesh.Exact("Family Practice" OR "General Practice" OR "Primary Health Care") OR "Primary Health Care" OR "Primary Healthcare" OR "General Practice" OR ("general practitioner" OR "general practitioners")) AND (mesh.Exact("COVID-19") OR "COVID-19" OR "Coronavirus" OR "corona*")

Additional limits - Date: After 01 December 2019

Bottom of Form

*Search Strategy for PDQ-Evidence.*

(title:((title:(video consultation) OR abstract:(video consultation)) OR (title:(e-consultation) OR abstract:(e-consultation)) OR (title:(e-health) OR abstract:(e-health)) OR (title:(ehealth) OR abstract:(ehealth)) OR (title:(telephone) OR abstract:(telephone)) OR (title:(alternative models of care) OR abstract:(alternative models of care)) OR (title:(virtual consult) OR abstract:(virtual consult)) OR (title:(Text Messaging) OR abstract:(Text Messaging)) OR (title:(telemedicine) OR abstract:(telemedicine)) OR (title:(telehealth) OR abstract:(telehealth)) OR (title:(remote consult*) OR abstract:(remote consult*)) OR (title:(tele*) OR abstract:(tele*)) OR (title:(videoconference) OR abstract:(videoconference)) OR (title:(video conference) OR abstract:(video conference)) OR (title:(digital health) OR abstract:(digital health))) OR abstract:((title:(video consultation) OR abstract:(video consultation)) OR (title:(e-consultation) OR abstract:(e-consultation)) OR (title:(e-health) OR abstract:(e-health)) OR (title:(ehealth) OR abstract:(ehealth)) OR (title:(telephone) OR abstract:(telephone)) OR (title:(alternative models of care) OR abstract:(alternative models of care)) OR (title:(virtual consult) OR abstract:(virtual consult)) OR (title:(Text Messaging) OR abstract:(Text Messaging)) OR (title:(telemedicine) OR abstract:(telemedicine)) OR (title:(telehealth) OR abstract:(telehealth)) OR (title:(remote consult*) OR abstract:(remote consult*)) OR (title:(tele*) OR abstract:(tele*)) OR (title:(videoconference) OR abstract:(videoconference)) OR (title:(video conference) OR abstract:(video conference)) OR (title:(digital health) OR abstract:(digital health)))) AND (title:((title:(primary healthcare) OR abstract:(primary healthcare)) OR (title:(primary health care) OR abstract:(primary health care)) OR (title:(general practitioner) OR abstract:(general practitioner)) OR (title:(family practice) OR abstract:(family practice)) OR (title:(general practice) OR abstract:(general practice)) OR (title:(general medicine) OR abstract:(general medicine))) OR abstract:((title:(primary healthcare) OR abstract:(primary healthcare)) OR (title:(primary health care) OR abstract:(primary health care)) OR (title:(general practitioner) OR abstract:(general practitioner)) OR (title:(family practice) OR abstract:(family practice)) OR (title:(general practice) OR abstract:(general practice)) OR (title:(general medicine) OR abstract:(general medicine))))

Limit: Publication Year Custom year range 2019 to 2021

Revised limit in search August 2022: Publication Year Custom year range 2020 to 2022
