# Supplementary material for: Visit Types in Primary Care With Telehealth Use During the COVID-19 Pandemic: Systematic Review
Source: JMIR Med Inform. 2022 Nov 28;10(11):e40469. doi: 10.2196/40469 (PMC9745650; doi:10.2196/40469)
Supplement: Multimedia Appendix 4 [file medinform_v10i11e40469_app4.docx]

# Appendix 4. Eligibility Criteria

## Table 4A. Eligibility Criteria for Systematic Review Article Inclusion

| **Criteria** | **Inclusion** | **Exclusion** |
| --- | --- | --- |
| Time period | December 2019 onwards (Post-COVID-19) | Before December 2019 (Pre-COVID-19) |
| Language | English | Not English |
| Telehealth format | Telehealth consultations in the format of; Telephone communication, video communication, text messaging, and email messaging | Telehealth services that do not reflect a consultation format (i.e., e-prescribing, patient portals, electronic health records, online health information, chat bots) |
| Population | Patients and Primary Health care providers (i.e., General practitioners) | Patients and Health care providers that are not within primary care settings (inclusive of medical students or trainees or specialists) |
| Context | Studies focused on a comparison of Telehealth consultations to face-to-face consultations within primary care settings | Studies not focused on a comparison of Telehealth consultations to face-to-face consultations within primary care settings (inclusive of triage calls/settings) |
| Concept/Objective | Studies focused on virtual care consultations and a review on health care-focused technological consultations. | Studies of technological interventions that do not explicitly focus on bi-directional provider-patient communication/consultation formats (e.g., patient portals that only focus on providing patients access to their health information, remote monitoring tools or programs without patient-provider communication functionality). |
| Outcomes (must include primary outcome) | Primary Outcome: Patient AND/OR General Practitioner Satisfaction Scale and Success of virtual care  Secondary Outcomes:  Feasibility, challenges of virtual care, engagement, accessibility, referral to in-person consultation. | Exclusion of studies that do not provide an assessment of consultation suitability to Telehealth/virtual care  Exclude studies focused on ‘macro’ outcomes (e.g., costs to the healthcare system, governments, public health overviews). |
| Comparison | Telehealth consultations to face-to-face interventions either through discussion or intervention.  Provides secondary comparison of suitability of Telehealth between different patient groups/visit types. | No comparison of telehealth interventions to face-to-face interventions or comparison between visit types/patient groups. |
| Perspective | Patients and/or primary healthcare worker’s perspective  Inclusive of expert opinion. | No presentation of patients and/or primary healthcare worker’s perspective or expert opinion. |
| Study design/format | Studies that used qualitative, quantitative or both approaches will be included  Literature reviews, systematic reviews, meta-analyses, scoping reviews, realist reviews, critical interpretive syntheses, opinion papers, commentaries, editorial reviews, primary research studies. | Study protocols |
