# Supplementary material for: Visit Types in Primary Care With Telehealth Use During the COVID-19 Pandemic: Systematic Review
Source: JMIR Med Inform. 2022 Nov 28;10(11):e40469. doi: 10.2196/40469 (PMC9745650; doi:10.2196/40469)
Supplement: Multimedia Appendix 5 [file medinform_v10i11e40469_app5.docx]

# Appendix 5. Data Extraction Template

*Adapted from Joanna Briggs Institute data abstraction formation and Fujioka et al., (2020) JMIR research protocols; 9(12): e22947 intended for use for data extraction methods.*

## Table 5A. Data Extraction Template

| Title & Authors | | Title:  In-Text Citation: |
| --- | --- | --- |
| Methods | | Description as stated in text |
|  | Study Design |  |
|  | Study Setting |  |
|  | Study Objectives/ Outcomes of Interest |  |
|  | Inclusion Criteria |  |
| Virtual Care | | Description as stated in text |
|  | Description of technology used |  |
|  | Categorisation of technology |  |
|  | Notes |  |
| Population | | Description as stated in text |
|  | Describe characteristics of study population (I.e., what makes group underserved) |  |
| Findings | | Description as stated in text |
|  | Types of Visit types |  |
|  | Facilitators of virtual care |  |
|  | Barriers of virtual care |  |
|  | Notes |  |
| Other | | Description as stated in text |
|  | Key Conclusions |  |
|  | Notes |  |
