# Supplementary material for: Visit Types in Primary Care With Telehealth Use During the COVID-19 Pandemic: Systematic Review
Source: JMIR Med Inform. 2022 Nov 28;10(11):e40469. doi: 10.2196/40469 (PMC9745650; doi:10.2196/40469)
Supplement: Multimedia Appendix 6 [file medinform_v10i11e40469_app6.docx]

# Appendix 6. Risk and Bias Critical Appraisal

The grading levels of evidence and GRADE scoring was determined using the Clinical Information Access Portal NSW Government Grading levels of evidence [39]. Risk and bias assessment highlight the lack of rigorous evidence available as there is an absence of systematic reviews or randomised controlled study designs. The data collected relies on semi-structured/structured interviews and cross-sectional surveys that can contain reporter or interviewer bias, which reduces the validity of results. However, thematic analysis based upon established frameworks, combined with similar findings shared amongst the individual studies, improves their recommendations’ reliability.

## Table 6A. Components used in the grading of the quality evidence and the strength of recommendations

| **Category** | **Grade** | **Description** |
| --- | --- | --- |
| Quality of Evidence | A | Evidence from high-quality randomised controlled trial (RCT) or systematic reviews/meta-analysis |
|  | B | Evidence from defective RCT, low-quality systematic reviews/meta-analyses, or high-quality observational studies |
|  | C | Evidence from non-randomised, case-control, or other observational studies |
|  | D | Expert individual opinion |
| Strength of Recommendation | 1 | Strong recommendation |
|  | 2 | Weak recommendation |
|  | 3 | No specific recommendation (insufficient evidence on which to formulate a recommendation) |

*Note: Components of GRADE scoring determined using (Jiang et al., 2018) [40]*

## Table 6B. Breakdown of included article by level of evidence, study design, Quality of Evidence grade and strength of recommendation (GRADE rating), MMAT scoring, and JBI appraisal tool scoring

| **Source (year, country)** | **Level of Evidence** | **Study Design** | **Quality of Evidence grade** | **Strength of recommendation** | **MMAT scoring** | **JBI Appraisal Tool** |
| --- | --- | --- | --- | --- | --- | --- |
| Gabrielsson-Jarhult, Kjellstrom, and Josefsson (2021, Sweden) | III | Mixed Methods Study | C | 1 | 100% | N/A |
| Mozes et al. (2022, Israel) | III | Mixed Methods Study | C | 1 | 86% | N/A |
| Murphy et al. (2021, United Kingdom) | III | Mixed Methods Study | C | 1 | 80% | N/A |
| Imlach et al. (2020, New Zealand) | III | Mixed Methods Study | C | 1 | 60% | N/A |
| Johnsen et al. (2021, Norway) | IV | Cross Sectional Survey | C | 2 | 80% | N/A |
| Hasani et al. (2020, Oman) | IV | Cross Sectional semi-structured interviews | C | 2 | 100% | N/A |
| Javanparast et al. (2021, Australia) | IV | Cross-Sectional semi-structured interviews | C | 2 | 70% | N/A |
| Schwelberger et al. (2020, United States of America) | IV | Cross Sectional Survey | C | 2 | 80% | N/A |
| Gomez et al. (2021, United States of America) | IV | Cross sectional semi-structured interviews | C | 2 | 60% | N/A |
| Grossman et al. (2020, Israel) | IV | Cross Sectional Survey | C | 2 | 40% | N/A |
| Jabbarpour et al. (2021, United States of America) | IV | Cross Sectional Survey | C | 2 | 60% | N/A |
| Jetty et al. (2021, United States of America) | IV | Cross Sectional Survey | C | 2 | 40% | N/A |
| De Guzman et al. (2022, Australia) | IV | Qualitative study semi-structured interviews | C | 2 | 60% | N/A |
| Due et al. (2021, Denmark) | IV | Qualitative study semi-structured interviews | C | 2 | 60% | N/A |
| Assing Hvidt et al. (2021, Denmark) | IV | Qualitative study structured interviews | C | 2 | 60% | N/A |
| Manksi-Nankervis et al. (2021, Australia) | IV | Cross Sectional Survey | C | 2 | 60% | N/A |
| Van de Poll-Franse et al. (2021, Netherlands) | IV | Cross Sectional Survey | C | 2 | 60% | N/A |
| RACGP (2021, Australia) | V | Guideline | D | 1 | N/A | N/A |
| MBS (2021, Australia) | V | Guideline | D | 1 | N/A | N/A |

*Note: The grading levels of evidence and GRADE scoring was determined using the following sources: Clinical Information Access Portal NSW Government Grading levels of Evidence [39]*

*‘N/A’ indicates scoring tool was ‘not applicable’ to the included articles study design.*

## Table 6C. **Mixed Methods Appraisal Tool (Version 2018) Scoring Sheets*:**

| Category of study designs | Methodological quality criteria | Responses | | | |
| --- | --- | --- | --- | --- | --- |
|  |  | Yes | No | Can’t Tell | Comments |
| Screening questions (for all types) | S1. Are there clear research questions? |  |  |  |  |
|  | S2. Do the collected data allow to address the research question? |  |  |  |  |
|  | *Further appraisal may not be feasible or appropriate when the answer is ‘No’ or ‘Can’t tell’ to one or both screening questions* | | | | |
| 1. Qualitative | 1.1. Is the qualitative approach appropriate to answer the research question? |  |  |  |  |
|  | 1.2. Are the qualitative data collection methods adequate to address the research question? |  |  |  |  |
|  | 1.3. Are the findings adequately derived from the data? |  |  |  |  |
|  | 1.4. Is the interpretation of results sufficiently substantiated by data? |  |  |  |  |
|  | 1.5. Is there coherence between qualitative data sources, collection, analysis, and interpretation? |  |  |  |  |
| 2. Quantitative randomised controlled trials | 2.1. Is randomization appropriately performed? |  |  |  |  |
|  | 2.2. Are the groups comparable at baseline? |  |  |  |  |
|  | 2.3. Are there complete outcome data? |  |  |  |  |
|  | 2.4. Are outcome assessors blinded to the intervention provided? |  |  |  |  |
|  | 2.5 Did the participants adhere to the assigned intervention? |  |  |  |  |
| 3. Quantitative non-randomised | 3.1. Are the participants representative of the target population? |  |  |  |  |
|  | 3.2. Are measurements appropriate regarding both the outcome and intervention (or exposure)? |  |  |  |  |
|  | 3.3. Are there complete outcome data? |  |  |  |  |
|  | 3.4. Are the confounders accounted for in the design and analysis? |  |  |  |  |
|  | 3.5. During the study period, is the intervention administered (or exposure occurred) as intended? |  |  |  |  |
| 4. Quantitative descriptive | 4.1. Is the sampling strategy relevant to address the research question? |  |  |  |  |
|  | 4.2. Is the sample representative of the target population? |  |  |  |  |
|  | 4.3. Are the measurements appropriate? |  |  |  |  |
|  | 4.4. Is the risk of nonresponse bias low? |  |  |  |  |
|  | 4.5. Is the statistical analysis appropriate to answer the research question? |  |  |  |  |
| 5. Mixed Methods | 5.1. Is there an adequate rationale for using a mixed method design to address the research question? |  |  |  |  |
|  | 5.2. Are the different components of the study effectively integrated to answer the research question? |  |  |  |  |
|  | 5.3. Are the outputs of the integration of qualitative and quantitative components adequately interpreted? |  |  |  |  |
|  | 5.4. Are divergences and inconsistencies between quantitative and qualitative results adequately addressed? |  |  |  |  |
|  | 5.5. Do the different components of the study adhere to the quality criteria of each tradition of the methods involved? |  |  |  |  |

**Note: Included literature that are from grey literature sources, systematic reviews, commentary, or opinion pieces are excluded from MMAT scoring.*

## Table 6D. **Joanna Briggs Institute scoring Sheets (**[**https://jbi.global/sites/default/files/2019-05/JBI_Critical_Appraisal-Checklist_for_Systematic_Reviews2017_0.pdf**](https://jbi.global/sites/default/files/2019-05/JBI_Critical_Appraisal-Checklist_for_Systematic_Reviews2017_0.pdf)**)** Scoring Sheet

| Question | Yes | No | Unclear | Not Applicable |
| --- | --- | --- | --- | --- |
| Is the review question clearly and explicitly stated? |  |  |  |  |
| Were the inclusion criteria appropriate for the review question? |  |  |  |  |
| Was the search strategy appropriate? |  |  |  |  |
| Were the sources and resources used to search for studies adequate? |  |  |  |  |
| Were the criteria for appraising studies appropriate? |  |  |  |  |
| Was critical appraisal conducted by two or more reviewers independently? |  |  |  |  |
| Were there methods to minimize errors in data extraction? |  |  |  |  |
| Were the methods used to combine studies appropriate? |  |  |  |  |
| Was the likelihood of publication bias assessed? |  |  |  |  |
| Were recommendations for policy and/or practice supported by the reported data? |  |  |  |  |
| Were the specific directives for new research appropriate? |  |  |  |  |

Overall appraisal: E.g. High, include

References:

39. Guyatt GH, Oxman AD, Schünemann HJ, et al. GRADE guidelines: a new series of articles in the journal of clinical epidemiology. J Clin Epidemiol 2011; 64: 380-382. 2010/12/28. doi: <https://doi.org/10.1016/j.jclinepi.2010.09.011>

40. Jiang M, Xie YQ, Xie JX, Zou XW, Lai KF. Methodology for development of the Chinese evidence-based Clinical Practice Guideline of the Diagnosis and Management of Cough. Journal of thoracic disease. 2018 Nov;10(11):6310.
