# Supplementary material for: Visit Types in Primary Care With Telehealth Use During the COVID-19 Pandemic: Systematic Review
Source: JMIR Med Inform. 2022 Nov 28;10(11):e40469. doi: 10.2196/40469 (PMC9745650; doi:10.2196/40469)
Supplement: Multimedia Appendix 7 [file medinform_v10i11e40469_app7.docx]

# Appendix 7. Visit Type Medicare Descriptions

The primary source for derived visit types and related definitions is the Medicare Benefits Scheme fact sheet information pages. These references are listed below:

29. MBS Changes factsheet - COVID-19 temporary MBS telehealth services. Department of Health, Australian Government. 2020 Sep 18. URL: http://www.mbsonline.gov.au/internet/mbsonline/publishing.nsf/Content/ 0C514FB8C9FBBEC7CA25852E00223AFE/$File/ Factsheet-COVID-19-Bulk-billed-MBS%20telehealth-Services-Overarching-17.09.2020.pdf [accessed 2021-03-03]

41. Medicare benefits schedule book operating from January 1 2020. Department of Health, Australian Government. 2021 Jul 21. URL: http://www.mbsonline.gov.au/internet/mbsonline/publishing.nsf/Content/ 8F3FA58ED97DCA35CA2584BE00111151/$File/202001-MBS%2017Jan2020.pdf [accessed 2021-07-23]

42. COVID-19 Telehealth MBS items. MBS Online. Phillip, Australia: Department of Health and Ageing, Australian Government; 2020 Aug 13. URL: http://www.mbsonline.gov.au/internet/mbsonline/publishing.nsf/Content/ news-2020-03-29-latest-news-March [accessed 2021-07-23]

43. Chronic Disease Management - Provider Information. The Department of Health and Aged Care. Phillip, Australia: Department of Health and Aged Care, Australian Government; 2016 Sep 2. URL: https://www1.health.gov.au/internet/ main/publishing.nsf/Content/mbsprimarycare-factsheet-chronicdisease.htm [accessed 2021-07-23]

***Chronic condition management:***

A chronic medical condition/disease and related visit type is defined as with the following description:

A chronic medical condition is one that has been (or is likely to be) present for six months or longer, for example, asthma, cancer, cardiovascular disease, diabetes, musculoskeletal conditions, and stroke. There is no list of eligible conditions. However, these items are designed for patients who require a structured approach to their care and to enable GPs to plan and coordinate the care of patients with complex conditions requiring ongoing care from a multidisciplinary team.

There are six CDM items that provide rebates for GPs to manage chronic or terminal medical conditions by preparing, coordinating, reviewing, or contributing to CDM plans.

***Mental health management:***

A Mental Health treatment/review visit type is defined as with the following description:

The GP Mental Health Treatment items incorporate a model for best practice primary health treatment of patients with mental disorders, including patients with both chronic or non-chronic disorders, that comprises:

· Assess and plan;

· Provide and/or refer for appropriate treatment and services;

· Review and ongoing management as required.

***Existing patients (acute or existing concern):***

Derived from the MBS Items for Standard GP consultations covering GP attendance for an obvious problem up to 40 minutes. Inclusive of existing or new concerns raised during consultations. Sub-sectioned for the means of this systematic review to define existing patients of the General Practice so that medical records are available, and a pre-existing patient-provider relationship is present.

***New patients (acute or existing concern):***

Derived from the MBS Items for Standard GP consultations covering GP attendance for an obvious problem up to 40 minutes. Inclusive of existing or new concerns raised during consultations. Sub-sectioned for the means of this systematic review to define new patients of the General Practice so that no medical records are available and there is no pre-existing patient-provider relationship present.

***Medication management:***

Derived from all Medicare category groups with the following subset activity defined rendering attracting of the Medicare item. The following are covered when there is a requirement for:

1. Medication recommendations Provide recommendations for immediate management, including the alternatives or options. This should include doses, expected response times, adverse effects and interactions, and a warning of any contra-indicated therapies.
2. Assess medications (including non-prescription medicines taken by the patient, prescriptions from other doctors, medications prescribed but not taken, interactions, side effects and review of indications); - Advise carers of the common side effects and interactions. - Consider the need for a formal medication review.
3. A Domiciliary Medication Management Review (DMMR) (Item 900), also known as Home Medicines Review, is intended to maximise an individual patient's benefit from their medication regimen and prevent medication-related problems through a team approach, involving the patient's GP and preferred community pharmacy or accredited pharmacist.
4. Development of a written medication management plan following discussion with the patient; and
5. provide the written medication management plan to a community pharmacy chosen by the patient

***Post-test results follow-up:***

Derived from all Medicare category groups with the following subset activity defined rendering attracting of the Medicare item. The following are covered when there is a requirement for:

1. any relevant recent diagnostic test results
2. making an overall assessment of the patient's risk factors and of the results of relevant examinations and investigations
3. review of initial presenting problems and results of diagnostic investigations
4. the results of other laboratory tests performed in the same episode meet the requirement/s as stipulated in the item descriptor; or
5. the results of laboratory tests that meet the requirement/s as stipulated in the item descriptor are supplied on the request form

This visit type also covers Medicare category specific descriptions such as the followings that are relevant to specific health conditions/concerns:

1. the reviewing of neuroimaging for the monitoring of a tumour or lesion and discussion of the results with the patient (e.g., meningiomaglioma, spinal cord tumour)
2. provision to the patient's preferred community pharmacy or accredited pharmacist, of relevant clinical information, by the medical practitioner for each individual patient, covering the patient's diagnosis, relevant test results and medication history, and current prescribed medications
3. the results of breast MRI imaging may alter treatment planning

***Post-discharge follow-up:***

Derived from all Medicare category groups with the following subset activity defined rendering attracting of the Medicare item. The following are covered when there is a requirement for:

1. multidisciplinary discharge care plan (prepared for a resident by another provider before the resident is discharged from a hospital or an approved day-hospital facility, or to a review of such a plan prepared by another provider)
2. significant change in the patient's condition or medication regimen requiring a new DMMR (specifically following recent discharge from hospital involving significant changes in medication)
3. A medical service rendered to a patient but prior to admission or subsequent to discharge
4. Private in-patients of a hospital (including private in-patients who are residents of aged care facilities) being discharged from hospital.
