# Supplementary material for: Visit Types in Primary Care With Telehealth Use During the COVID-19 Pandemic: Systematic Review
Source: JMIR Med Inform. 2022 Nov 28;10(11):e40469. doi: 10.2196/40469 (PMC9745650; doi:10.2196/40469)
Supplement: Multimedia Appendix 8 [file medinform_v10i11e40469_app8.docx]

# Appendix 8. Supporting Evidence of Suitability of Telehealth for each Visit Type during COVID-19

## Table 8A. Benefits Findings of Telehealth by Outcome Measures of Included Studies per Visit Type from the Patient’s Perspective

| **Visit Type** | **Benefits Findings** | **Supportive Evidence** |
| --- | --- | --- |
| **Chronic Condition Management/Review** | **Access to Care:**   - *Timely access and more frequent access to care for at-risk patient groups:* Primary care providers can provide timely and more frequent access to chronic conditions and shielding patients to primary care services [1, 24] | **Access to Care:**   - Shielding patients are able to access safe care during the pandemic with GP consultations easily increased in April to July 2020 compared with April to July 2019 (Incidence rate ratio = 1.09, P = 0.004) [24] |
|  | **Effectiveness:**   - *Activities observed in Chronic condition management visits are suitable to Telehealth:* Activities observed such as routine check-ups and chronic condition medication renewal identified as suitable for Telehealth by patients [1] | **Effectiveness:**  Moreover, under the given circumstances, we found that VCs were suitable for a variety of mental health problems, along with other chronic or complex issues such as chronic pain, tiredness, sleeping problems, follow-up of established cancer treatment, and administrative purposes [1] |
|  | **Experience:**   - *High patient satisfaction and willingness to engage with Telehealth:* High patient satisfaction and willingness to engage with Telehealth for chronic condition consultations without a physical examination or complex diseases [31] | **Experience:**   - “Absolutely, if I’m just doing that 6 or 12-monthly blood test thing, I’d be more than happy for him to mail me the paperwork and ring up for a report - no dramas. It’s only when I have something that I feel I need to see him about. (male, 73 yrs. old)” [31] |
|  | - ***Lower risk of COVID-19 transmission protects at-risk patient groups:*** High-risk patients (i.e., comorbidities, metastasised cancer, of child’s age or older age, concerns of COVID-19 transmission) were most suited to Telehealth [21, 26] | - High-risk patients with cancer or chronic conditions (917/4094, 22%) were more worried about getting infected with SARS-CoV-2 compared with norm participants (175/977, 18%) [21] |
|  | **Financial Impact/Cost:**   - *Suitable Medicare support available for this visit-type for Australian patients:* Chronic/Disability patients covered by Medicare when developing a treatment and management plan [28, 29] | **Financial Impact/Cost:**   - Consultation develops a treatment and management plan, which must include the following: an assessment and diagnosis of the patient’s condition; a risk assessment; treatment options and decisions; if necessary—medication recommendations with $138.70 rebate and 92145 item number [28] |
| **Existing patient (acute or existing concern). Inclusive of shielding patients** | **Access to Care:**   - N/A visit specific evidence | **Access to Care:**   - N/A visit specific evidence |
|  | **Effectiveness:**   - *Positive pre-existing patient-provider relationship suitable to Telehealth due to established understanding of patient history and existing concerns:* patient Telehealth reported to be effective for existing patients due to an established patient-provider relationship, except for cases requiring physical examination [24, 27] | **Effectiveness:**   - Clinicians rated (mean; SD) care-coordination (2.8;0.q7) and follow-up care (2.3;0.6) high when questioned their perceptions of usability and usefulness of telemedicine on a 4-point scale. [27] |
|  | - *Dependent on the severity of the problem:* Simple concerns are suitable for Telehealth [30] | - “If I don’t need anything serious, then why should I waste my time going to the PCP [physically]?” (2) [30] - “If there is a small problem, patients [tend] to prefer a phone visit…and prefer a video [call] if it’s more of a big problem.” (3) [30] |
|  | Experience:   - *Satisfying patient expectations due to pre-existing relationship with their clinician:* High satisfaction and reduced hesitancy for existing patients due to pre-existing patient-provider relationship [30] | Experience:   - “Of course, if I know the PCP already, it’s much easier to trust him from a distance.” (2) If it’s a doctor I already know - he knows me, I know him - so…you know, we already have ‘a common language’. Then, maybe it’s easier for me…that’s why I’ve already had a few video visits.” (3) [30] - “For patients I know and have treated before – [meaning],I know his background, his diseases…[I feel comfortable] giving him digital service - either via video or online form.”(2) [30] |
|  | - *Satisfying patient expectations for simple consultations (i.e., concerns without need for a physical examination):* High patient satisfaction and willingness to engage with Telehealth for acute concerns without a physical examination or existing problems requiring general follow-up resulting [1] | - N/A visit specific evidence |
|  | - ***Lower risk of COVID-19 transmission for shielding patients:*** Shielding patients satisfied due to reduced risk of COVID-19 transmission and additional focus on high-risk patients during the pandemic [26] | - ‘Normalisation Process Theory model of remote consulting’ identifies a “focus on vulnerable: older, shielding patients and patients with mental health problems” as a “collective action” incorporated by Telehealth [26] |
|  | **Financial Impact/Cost:**   - N/A visit specific evidence | **Financial Impact/Cost:**   - N/A visit specific evidence |
| **Mental health/behavioural management/review** | **Access to Care:**   - *Reduced wait times (i.e., the remote nature makes consultations run more efficiently and quickly in comparison to FTFC):* Primary care providers can provide timely and more frequent access to mental health care services for patients using Telehealth [26, 27] | **Access to Care:**   - High-risk mental health patients were able to access safe care during the pandemic, with consultations easily increased in April to July 2020 compared with April to July 2019 for GPs (Incidence rate ratio = 1.06, P = 0.002) [26] |
|  | **Effectiveness:**   - N/A visit specific evidence | **Effectiveness:**   - N/A visit specific evidence |
|  | **Experience:**   - *Satisfying patient expectations of what they expected from the Telehealth consultation and their clinician when questioned in a survey:* Mental health/behavioural patients reported satisfaction was high, as well as a willingness to engage with Telehealth again in the future [1, 21] | **Experience:**   - Among patients with depression, included article Choi et al reported high levels of treatment acceptance in both groups, but patients in the video consultation group had a more favourable attitude towards treatment. [21] |
|  | **Financial Impact/Cost:**   - *Suitable Medicare support due to existing Medicare items available to cover costs for Australian patients*: GP Mental Health Treatment Plans and Counselling supported by Medicare (i.e., item number 92114) [28, 29] | **Financial Impact/Cost:**   - Telehealth attendance by a general practitioner to review a GP mental health treatment plan which the general practitioner or an associated general practitioner has prepared or to review a Psychiatrist Assessment and Management Plan covered by $73.95 rebate under item number 92114 [28] |
| **New patient (acute or existing concern). Inclusive of shielding patients.** | **Access to Care:**   - N/A visit specific evidence | **Access to Care:**   - N/A visit specific evidence |
|  | **Effectiveness:**   - N/A visit specific evidence | **Effectiveness:**   - N/A visit specific evidence |
|  | **Experience:**   - N/A visit specific evidence | **Experience:**   - N/A visit specific evidence |
|  | **Financial Impact/Cost:**   - *Suitable Medicare support due to existing Medicare items available to cover costs for Australian patients:* Telehealth Medicare cover for new patients, specifically for psychiatric consultations [29] | **Financial Impact/Cost:**   - Telehealth Medicare cover for psychiatrist, attendance, new patient (or has not received attendance in preceding 24 months), more than 45 minutes: 92437 Telehealth Item number respectively [29] |
| **Medication treatment/review (non-chronic condition)** | **Access to Care:**   - N/A visit specific evidence | **Access to Care:**   - N/A visit specific evidence |
|  | **Effectiveness:**   - N/A visit specific evidence | **Effectiveness:**   - N/A visit specific evidence |
|  | **Experience:**   - *Satisfying patients receiving prescriptions via Telehealth:* Patients reported feeling satisfied with receiving prescriptions via Telehealth [31] | **Experience:**  “Unless it’s urgent I reckon the phone would be just fine after this pandemic period, especially for something like prescriptions or simple things. (female, 54 yrs. Old)” [31] |
|  | **Financial Impact/Cost:**   - *Suitable Medicare support due to existing Medicare items available to cover costs for Australian patients:* Medication reviews and recommendation consultations Medicare supported [28, 29] | **Financial Impact/Cost:**   - E.g., Develops a treatment and management plan, which includes treatment options, decisions, and medication recommendations supported by a $138.70 rebate and 92142 item number [28] |
| **Post-test results follow-up** | **Access to Care:**   - *Confidentiality in providing test results:* Procedures are in place to ensure confidentiality via Telehealth consultations to aid buy-in and access to care for hesitant patients [23] | **Access to Care:**   - N/A visit specific evidence |
|  | **Effectiveness:**   - N/A visit specific evidence | **Effectiveness:**   - N/A visit specific evidence |
|  | **Experience:**   - N/A visit specific evidence | **Experience:**   - N/A visit specific evidence |
|  | **Financial Impact/Cost:**   - N/A visit specific evidence | **Financial Impact/Cost:**   - N/A visit specific evidence |
| **Post-discharge follow-up** | **Access to Care:**   - N/A visit specific evidence | **Access to Care:**   - N/A visit specific evidence |
|  | **Effectiveness:**   - N/A visit specific evidence | **Effectiveness:**   - N/A visit specific evidence |
|  | ***Experience:***   - N/A visit specific evidence | **Experience:**   - N/A visit specific evidence |
|  | **Financial Impact/Cost:**   - *Suitable Medicare support due to existing Medicare items available to cover costs for Australian patients:* Telehealth Medicare cover for post-discharge postnatal consultations conducted by an obstetrician or general practitioner [28, 29] | **Financial Impact/Cost:**   - Telehealth Medicare cover for post-discharge postnatal consultations*:* $62.90 and 91851 for the rebate and Item number, respectively [28] |

## Table 8B. Benefits Findings of Telehealth by Outcome Measures of Included Studies per Visit Type from the GP’s Perspective

| **Visit Type** | **Benefits Findings** | **Supporting Evidence** |
| --- | --- | --- |
| **Chronic condition management/review** | **Access to Care:**   - *Timely access and reduced risk to COVID-19:* Primary care providers can provide timely and more frequent access to chronic conditions and shielding patients to primary care services [1, 26, 27] | **Access to Care:**   - N/A visit specific evidence |
|  | **Effectiveness:**   - *Amendable for visit type patient-groups (e.g., due to ease in monitoring at risk patients, can conduct routine diabetic check-ups more conveniently):* Highly amendable and effective for chronic condition consultations patients due to ease in monitoring high-risk, complex patients (e.g., diabetic patients with routine check-ups) [1, 18, 22, 27] | **Effectiveness:**   - 75/106 (71%) rated VC suitability for complex issues (e.g., chronic pain, sleeping problems, fatigue) consultations to be better or the same as FTF [1] - 30/40 (75%) rated VC suitability for cancer follow-up consultations to be better or the same as FTF [1] - 60/114 (53%) rated VC suitability for endocrinology (e.g., diabetes, thyroid) consultations to be better or the same as FTF [1] - Chronic condition consultations for asthma, diabetes, and hypertension were 242/782 (31%), 284/887 (32%), and 1106/3567 (31%) Telehealth amendable [18] - “Chronic disease management, I think can be done much more virtually. And I think we’re headed in that direction” (P12) [22] - Clinicians rated (mean; SD) care-coordination (2.8;0.7), follow-up care (2.3;0.6), and chronic disease management (2.5;0.7) highest when questioned their perceptions of usability and usefulness of telemedicine on a 4-point scale. [27] |
|  | - *Amendable for visit type activities (e.g., symptom review, feedback, prescription refills, chronic disease management, and counselling):* **Activities common within this visit type are highly amendable to Telehealth (e.g.,** Symptom review, feedback, prescription refills, chronic disease management, education, and counselling) **[18]** | - “Symptom review, feedback, prescription refills, chronic disease management, education, and counselling may all be done via e-mail, text, and other” [18] |
|  | - ***Suitable in times where physical examinations are not required (e.g., health education or counselling):* Suitable for chronic condition consultations without a requirement for physicians to be present, including h**ealth education or counselling for asthma education, asthma action plan given to a patient, and diabetes education deemed suitable [20] | - Quoted statement: “Suitable for **chronic condition consultations without a requirement for physicians to be present, h**ealth education or counselling for asthma education, asthma action plan given to a patient, and diabetes education deemed suitable” [20] |
|  | - *Additional suitability if supported by at-home medical devices (e.g., blood pressure cuffs, glucometers):* Physicians also noted that medication adjustments for chronic conditions such as hypertension and diabetes could easily be done through telemedicine visits if patients have working devices at home to monitor their conditions (i.e., blood pressure cuffs, glucometers) [22] | - “I can even do diabetes follow up [with telemedicine] if I know that the patient has a good blood glucose monitor and is good about keeping data” (P7) [22] |
|  | **Experience:**   - *Remote nature of Telehealth favoured by high-risk patients at risk of poor COVID-19 symptoms if exposed:* High-risk patients (i.e., comorbidities, metastasised cancer, of child's age or older age, concerns of COVID-19 transmission) were most suited to Telehealth [21, 23, 26, 28, 29] | **Experience:**   - “Telephone consultation helped our patients with chronic conditions because those with co-morbidities are at higher risk to develop complications from COVID-19 and we are happy to keep them at home.” P1 [23] - “. . .surely it reduced the risk of contracting COVID-19 from the health center, especially in diabetic and asthmatic patients” P19 [23] |
|  | **Financial Impact/Cost:**   - *Suitable Medicare support due to existing Medicare items available to cover costs for Australian patients:* Chronic/Disability patients covered by Medicare when developing a treatment and management plan [28, 29] | **Financial Impact/Cost:**   - Consultation develops a treatment and management plan, which must include the following: an assessment and diagnosis of the patient’s condition; a risk assessment; treatment options and decisions; if necessary—medication recommendations with $138.70 rebate and 92145 item number [28] |
| **Existing patient (acute or existing concern). Inclusive of shielding patients** | **Access to Care:**   - N/A visit specific evidence | **Access to Care:**   - N/A visit specific evidence |
|  | **Effectiveness:**   - *Suitable pre-existing patient-provider relationship for Telehealth as clinicians know patient history:* Telehealth to be effective for existing patients due to an established patient-provider relationship, except for cases requiring physical examination [1, 23, 27] | **Effectiveness:**   - Clinicians rated (mean; SD) care-coordination (2.8;0.7), follow-up care (2.3;0.6), and chronic disease management (2.5;0.7) highest when questioned their perceptions of usability and usefulness of telemedicine on a 4-point scale. [27] - 1140/1866 (61%) rated VC as suitable for existing patients as better or same as FTF [1] - The suitability rate for existing patients for follow-up sessions (existing problem) was 1165/1919 (61%), compared to 544/1556 (35%) for new patient problems [1] - “It will work better with those with chronic conditions and for follow up” P2 [23] |
|  | - *Suitable for dermatological concerns (i.e., video and imaging technology aids patient assessment):* Telehealth deemed suitable for existing or acute dermatological concerns [1] | - N/A visit specific evidence |
|  | - *Minimal difference to consultation quality reported by clinicians in survey responses:* Primary care providers can provide quality care services using Telehealth [9] | - 'I reckon this is the truth that a lot of doctors weren’t going to really say, we could still do good quality care 90 to 95% of the time on telehealth.' (GP11) [9] |
|  | **Experience:**   - N/A visit specific evidence | **Experience:**   - N/A visit specific evidence |
|  | **Financial Impact/Cost:**   - N/A visit specific evidence | **Financial Impact/Cost:**   - N/A visit specific evidence |
| **Mental health/behavioural management/review** | **Access to Care:**   - N/A visit specific evidence | **Access to Care:**   - N/A visit specific evidence |
|  | **Effectiveness:**   - *Suitable to visit type due to predominately requiring discussion activities:* Clinicians reported Telehealth highly effective for mental/behavioural concern patients due to activities predominantly being in-depth discussion/counselling inclusive of follow-up [1, 20, 23] | **Effectiveness:**   - 509/684 (74%) clinicians rated VC suitability for mental illness/life stress consultations to be better or the same as FTF [1] |
|  | - *Suitable as physical presence is not required (i.e., depression screening or mental health counselling):* Physical presence is not required for depression screening, other mental health screening, or mental health counselling [20] | - N/A visit specific evidence |
|  | - *Suitable for patients with mild mental health issues:* The GPs considered video consultation suitable for patients with mild mental illness [1] | - N/A visit specific evidence |
|  | **Experience:**   - N/A visit specific evidence | **Experience:**   - N/A visit specific evidence |
|  | **Financial Impact/Cost:**   - *Suitable Medicare support due to existing Medicare items available to cover costs for Australian patients:* GP Mental Health Treatment Plans and Counselling supported by Medicare [28, 29] | **Financial Impact/Cost:**   - Telehealth attendance by a general practitioner to review a GP mental health treatment plan which the general practitioner or an associated general practitioner has prepared or to review a Psychiatrist Assessment and Management Plan covered by $73.95 rebate under item number 92114 [28] |
| **New patient (acute or existing concern). Inclusive of shielding patients.** | **Access to Care:**   - N/A visit specific evidence | **Access to Care:**   - N/A visit specific evidence |
|  | **Effectiveness:**   - *Suitable for existing concerns (i.e., diagnosed health concerns):* Existing concerns, irrespective of being new or existing patients, were suitable for Telehealth due to established diagnoses (e.g., skin conditions) [1, 27] | **Effectiveness:**   - 18% (33/183) and 54% (154/286) of musculoskeletal problems, and 20% (4/20) and 77% (39/51) for neurology disorders, acute versus existing problems respectively, were rated suitable to Telehealth [1] - Suitability of telemedicine for acute care (applicable to new patients) in intermediate telehealth use practices was 2.3 (0.5) out of a 4-point scale [27] |
|  | - *Suitable for simple acute concerns (e.g., simple disease presentations and symptoms with no need for physical examination):* There is rated suitability of telemedicine for acute care issues, often presented by new patients if simple (i.e., simple disease presentations and symptoms) deemed [23, 25, 27] | - N/A visit specific evidence |
|  | - *Suitable for dermatological concerns (i.e., video and imaging technology aids patient assessment):* Telehealth deemed suitable for existing or acute dermatological concerns [9] | - “They mentioned videoconference is … effective … for scenarios that require visual assessment, such as skin or throat issues. They emphasised the importance of considering clinical needs when choosing consultation mode” [9] |
|  | **Experience:**   - N/A visit specific evidence | **Experience:**   - N/A visit specific evidence |
|  | **Financial Impact/Cost:**   - *Suitable Medicare support due to existing Medicare items available to cover costs for Australian patients:* Telehealth Medicare cover for new patients, specifically for psychiatric consultations [29] | **Financial Impact/Cost:**   - Telehealth Medicare cover for psychiatrist, attendance, new patient (or has not received attendance in preceding 24 months), more than 45 minutes: 92437 Telehealth Item number respectively [29] |
| **Medication treatment/review (non-chronic condition)** | **Access to Care:**   - N/A visit specific evidence | **Access to Care:**   - N/A visit specific evidence |
|  | **Effectiveness:**   - *Suitable for prescription refills:* Prescription refills deemed to be suitable to Telehealth [18] | **Effectiveness:**   - “Symptom review, feedback, prescription refills, chronic disease management, education, and counselling may all be done via e-mail, text, and other” [18] |
|  | - *Suitable for simple consultations and health concerns (e.g., oral contraceptives as risk of adverse medical event are low):* Medication reviews and providing prescriptions can be completed simply for patients without complex physical examinations required (e.g., oral contraceptives) [23] | - “We can give pharmacy prescriptions and normal investigation results like urine and CBC. If the patient is stable and needs only a little bit of adjustment of medications, we can give this” P19 [23] |
|  | - *Ease in conducting medication reconciliations (e.g., patients can show their clinicians their medications via video or image sharing easily):* Medication reconciliations are easier to conduct via telemedicine because patients can share their medications easily and safely with their practitioners [1, 22] | - “Patients can show you their medications, read the labels” (P2) [22] |
|  | - *Suitable for prescription of established medications:* Telehealth is suitable for GPs providing prescriptions for existing medications [1] | - Prescription of established medication via VC was deemed “better or same” as FTFC in 220/408 (54%) of responses [1] |
|  | **Experience:**   - *Greater relief in not prescribing addictive drugs to at-risk patients:* GPs experience relief as it is easier to say no to prescribing addictive drugs to patients [22] | **Experience:**   - With Telehealth I feel comfortable telling a patient I am not going to give them their benzodiazepines or their pain medications. They have a video in front of them instead of the person’s right there yelling at me” (P10) [22] |
|  | **Financial Impact/Cost:**   - *Suitable Medicare support due to existing Medicare items available to cover costs for Australian patients:* Medication reviews and recommendation consultations Medicare supported [28, 29] | **Financial Impact/Cost:**   - Develops a treatment and management plan, which includes treatment options, decisions, and medication recommendations supported by a $138.70 rebate and 92142 item number [29] |
| **Post-test results follow-up** | **Access to Care:**   - *Confidential processes in place for providing test results (e.g., calling on their private number and using ID number confirmations):* Procedures are in place to ensure confidentiality via Telehealth consultations to aid buy-in and access to care for hesitant patients [23] | **Access to Care:**   - “. . .. about COVID results, we never give positive results for incoming calls; we will call patients on their private numbers and ask about ID number” P15. [23] |
|  | **Effectiveness:**   - *High suitability of Telehealth consultations due to time dedicated to discussions and not physical examinations:* High suitability of Telehealth consultations focused on counselling, such as review of laboratory results, as it promotes deeper discussions [22, 23] | **Effectiveness:**   - “I suddenly have 6 or 7 or 8 or 9 minutes that I would have spent on physical examination to devote toward discussion” (P3) [22] - “We can give pharmacy prescriptions and normal investigation results like urine and CBC. If the patient is stable and needs only a little bit of adjustment of medications, we can give this” P19 [23] |
|  | - *Improved ability for follow-up and sharing information (e.g., out-of-clinic communication channels to assist patients):* Telehealth enables increased follow up and ability to share information, which is important for post-test results discussion patients or patients understanding of diagnoses provided [26] | - “I’ll write quite detailed texts to patients who I’ve just spoken to, saying, “You might want to try this website” […] all you have to do is cut-and-paste a link and some people then have immediately got the website on their phone.” (GP, HC19, Round 2) [26] |
|  | - *Existing procedural processes for sharing lab results remotely and with consideration of confidentiality:* Procedures in place for confidentiality protects patients receiving stigmatised or sensitive test results [23] | - “. . .we never give lab results to another person, especially for abnormal results or for something sensitive like hepatitis screening” P14, [23] |
|  | **Experience:**   - N/A visit specific evidence | **Experience:**   - N/A visit specific evidence |
|  | **Financial Impact/Cost:**   - N/A visit specific evidence | **Financial Impact/Cost:**   - N/A visit specific evidence |
| **Post-discharge follow-up** | **Access to Care:**   - N/A visit specific evidence | **Access to Care:**   - N/A visit specific evidence |
|  | **Effectiveness:**   - *Improving ability to conduct more frequent and same-day visits for ante-natal or post-discharge patients:* Telehealth enables increased follow up, which is important for post-discharge/procedure patients (e.g., post-surgery wound care follow-up) [23, 26] | **Effectiveness:**   - “Nurses will follow up patients for vaccination, ante-natal care, follow-up COVID-19 low-risk patients, and schedule ANC visit and scan in the same day.” P3 [23] |
|  | **Experience:**   - N/A visit specific evidence | **Experience:**   - N/A visit specific evidence |
|  | **Financial Impact/Cost:**   - *Suitable Medicare support due to existing Medicare items available to cover costs for Australian patients:* Telehealth Medicare cover for post-discharge postnatal consultations conducted by an obstetrician or general practitioner [28, 29] | **Financial Impact/Cost:**   - Telehealth Medicare cover for post-discharge postnatal consultations*:* $62.90 and 91851 for the rebate and Item number, respectively [28] |

## Table 8C. Drawback Findings of Telehealth by Outcome Measures of Included Studies per Visit Type from the Patient’s Perspective

| **Visit Type** | **Drawback Findings** | **Supporting Evidence** |
| --- | --- | --- |
| **Chronic condition management/review** | **Access to Care:**   - *Hesitant patients to use Telehealth due to uncertainty of Telehealth encounters, which further reduces at-risk patients (e.g., cancer patients) access to care:* Concerns about not reaching at-risk patients in need of care due to patient hesitancy to attend Telehealth consultations [21] | **Access to Care:**   - Cancer patients (852/4068, 20.9%) and norm patients (218/979, 22.3%) reported that the COVID-19 crisis made them contact their general practitioner less quickly when they had physical complaints or concerns due to hesitancy of Telehealth consultations [21] |
|  | **Effectiveness:**   - *Not suitable for patients with complex problems (e.g., co-morbidity, low mobility, complex social issues, low hearing and vision, and cognitive impairment):* Telehealth is not suited to chronic condition patients with complex problems (e.g., co-morbidity) [30] | **Effectiveness:**   - “If it’s all sorts of small things, let’s say related to the skin - skin diseases, some kind of fungal infection, I prefer to see my PCP digitally*.* [But], if I had a concern about skin cancer, I might have gone to the PCP to see him [frontally]” (1) [30] |
|  | **Experience:**   - N/A visit specific evidence | **Experience:**   - N/A visit specific evidence |
|  | **Financial Impact/Cost:**   - N/A visit specific evidence | **Financial Impact/Cost:**   - N/A visit specific evidence |
| **Existing patient (acute or existing concern). Inclusive of shielding patients** | **Access to Care:**   - N/A visit specific evidence | **Access to Care:**   - N/A visit specific evidence |
|  | **Effectiveness:**   - N/A visit specific evidence | **Effectiveness:**   - N/A visit specific evidence |
|  | **Experience:**   - N/A visit specific evidence | **Experience:**   - N/A visit specific evidence |
|  | **Financial Impact/Cost:**   - N/A visit specific evidence | **Financial Impact/Cost:**   - N/A visit specific evidence |
| **Mental health/behavioural management/review** | **Access to Care:**   - N/A visit specific evidence | **Access to Care:**   - N/A visit specific evidence |
|  | **Effectiveness:**   - *Differing views on the usefulness of Telehealth for mental health issues felt by patients:* Telehealth not seen as beneficial for Telehealth due to lack of in-person contact [31] | **Effectiveness:**   - “If you had mental health problems that would probably be a difficult one to talk about over the phone, you might need to speak to somebody.’ (female, 68 yrs. old), another participant stated: ‘I see the benefit in the mental health space for argument’s sake where people are able to talk…to just be there privately in your own room with your computer talking to the person, it does assist a lot’ (male, 59 yrs. old)” [31] |
|  | **Experience:**   - N/A visit specific evidence | **Experience:** |
|  | **Financial Impact/Cost:**   - N/A visit specific evidence | **Financial Impact/Cost:**   - N/A visit specific evidence |
| **New patient (acute or existing concern). Inclusive of shielding patients.** | **Access to Care:**   - N/A visit specific evidence | **Access to Care:**   - N/A visit specific evidence |
|  | **Effectiveness:**   - N/A visit specific evidence | **Effectiveness:**   - N/A visit specific evidence |
|  | **Experience:**   - N/A visit specific evidence | **Experience:** |
|  | **Financial Impact/Cost:**   - N/A visit specific evidence | **Financial Impact/Cost:**   - N/A visit specific evidence |
| **Medication treatment or review (non-chronic condition)** | **Access to Care:**   - N/A visit specific evidence | **Access to Care:**   - N/A visit specific evidence |
|  | **Effectiveness:**   - *Concerns when physical examinations are necessary (e.g., checking for infections when prescribing antibiotics):* Patients are concerned of the effectiveness of Telehealth in times where physical examinations are needed to make changes to medications [30] | **Effectiveness:**   - Because my doctor knows me, I could say, “I need antibiotics or prescription”, and he says, “Yeah, okay”… but if you are talking by phone with a doctor that hasn’t seen you or doesn’t know you. That’s a big difference, isn’t it? (female, 68 yrs. old) [30] |
|  | **Experience:**   - N/A visit specific evidence | **Experience:**   - N/A visit specific evidence |
|  | **Financial Impact/Cost:**   - N/A visit specific evidence | **Financial Impact/Cost:**   - N/A visit specific evidence |
| **Post-test results follow-up** | **Access to Care:**   - N/A visit specific evidence | **Access to Care:**   - N/A visit specific evidence |
|  | **Effectiveness:**   - N/A visit specific evidence | **Effectiveness:**   - N/A visit specific evidence |
|  | **Experience:**   - N/A visit specific evidence | **Experience:** |
|  | **Financial Impact/Cost:**   - N/A visit specific evidence | **Financial Impact/Cost:**   - N/A visit specific evidence |
| **Post-discharge follow-up** | **Access to Care:**   - N/A visit specific evidence | **Access to Care:** |
|  | **Effectiveness:**   - N/A visit specific evidence | **Effectiveness:**   - N/A visit specific evidence |
|  | **Experience:**   - N/A visit specific evidence | **Experience:**   - N/A visit specific evidence |
|  | **Financial Impact/Cost:**   - N/A visit specific evidence | **Financial Impact/Cost:**   - N/A visit specific evidence |

## Table 8D. Drawback Findings of Telehealth by Outcome Measures of Included Studies per Visit Type from the GP’s Perspective

| **Visit Type** | **Drawback Findings** | **Supporting Evidence** |
| --- | --- | --- |
| **Chronic condition management/review** | **Access to Care:**   - N/A visit specific evidence | **Access to Care:**   - N/A visit specific evidence |
|  | **Effectiveness:**   - *Lower quality score rating of Telehealth consultations in comparison to FTFC:* FTF consultations scored higher than VC and TC related to the difference in length of time and discussions [9] | **Effectiveness:**   - 'Limiting it to brief consultations, not longer consultations, not mental health consultations, not chronic disease care planning consultation will end up disadvantaging the patient.' (GP54) [9] |
|  | - *Additional precautions required for at-risk patients* *(e.g., cancer patients or co-morbidities at risk of adverse medical events*): Additional care is required for at-risk patients such as cancer patients or patients with co-morbidities when using Telehealth due to risk of adverse medical event [1, 25] | - “Particular caution must be shown in situations where patient safety might be at risk, including the evaluation of potential malignancy” [1] |
|  | **Experience:**   - N/A visit specific evidence | **Experience:**   - N/A visit specific evidence |
|  | **Financial Impact/Cost:**   - N/A visit specific evidence | **Financial Impact/Cost:**   - N/A visit specific evidence |
| **Existing patient (acute or existing concern). Inclusive of shielding patients** | **Access to Care:**   - N/A visit specific evidence | **Access to Care:**   - N/A visit specific evidence |
|  | **Effectiveness:**   - *Lower quality score rating of Telehealth consultations in comparison to FTFC:* FTF consultations scored higher than VC and TC on the consultation-quality item for an explanation of diagnosis, important for acute and existing concerns [9] | **Effectiveness:**   - 'Limiting it to brief consultations, not longer consultations, not mental health consultations, not chronic disease care planning consultation will end up disadvantaging the patient.' (GP54) [9] |
|  | **Experience:**   - N/A visit specific evidence | **Experience:**   - N/A visit specific evidence |
|  | **Financial Impact/Cost:**   - N/A visit specific evidence | **Financial Impact/Cost:**   - N/A visit specific evidence |
| **Mental health/behavioural management/review** | **Access to Care:**   - N/A visit specific evidence | **Access to Care:**   - N/A visit specific evidence |
|  | **Effectiveness:** | **Effectiveness:** |
|  | - *Difficult with unstable mental status due to increased risk of adverse events and harder to engage (e.g., suicidal patients):* Patients with an unstable mental status are note suited to Telehealth due to risk of adverse medical event [1] |  |
|  | **Experience:**   - N/A visit specific evidence | **Experience:**   - N/A visit specific evidence |
|  | **Financial Impact/Cost:**   - N/A visit specific evidence | **Financial Impact/Cost:**   - N/A visit specific evidence |
| **New patient (acute or existing concern). Inclusive of Shielding patients.** | **Access to Care:**   - N/A visit specific evidence | **Access to Care:**   - N/A visit specific evidence |
|  | **Effectiveness:**   - *Not suitable for any consultations requiring a new diagnosis:* Any consultations that involve diagnostic elements, which is often involved in new patient consultations, are deemed less suitable to Telehealth formats [1, 22] | **Effectiveness:**   - The suitability rate was 35% (544/1556) when the patient presented a new problem. For example, the suitability of VC for skin disorders was 30% (69/234) for a new problem and 44% (29/66) for a previously discussed problem [1] - “It is hard to replace the in-person [visit], even if you did not need to examine the patient, because you appreciate a lot of the nuances, how the patient’s acting in the office, and stuff that you cannot appreciate over telehealth” (P5) [22] |
|  | - *Not suited to complex symptom presentations that require physical examinations (e.g., chest pain, stomach pain, potential new cancer):* VC was considered less suitable in situations involving complex symptom presentations [1] | - VC was considered less suitable in situations involving acute chest pain, stomach pain, and fear/investigation of a potential new cancer [1] |
|  | - *Poor suitability without pre-existing patient-provider relationship (i.e., pre-existing knowledge of patient history and existing rapport):* VC was considered less suitable in situations with new patients due to not knowing patient history and concerns in-depth [1] | - When the patient was “unknown,” the corresponding suitability rate dropped to 32% (87/274) [1] |
|  | **Experience:**   - N/A visit specific evidence | **Experience:** |
|  | **Financial Impact/Cost:**   - N/A visit specific evidence | **Financial Impact/Cost:**   - N/A visit specific evidence |
| **Medication treatment/review (non-chronic condition)** | **Access to Care:**   - N/A visit specific evidence | **Access to Care:**   - N/A visit specific evidence |
|  | **Effectiveness:**   - *Not suited to antibiotic prescription when clinicians were surveyed:* Telehealth is not suited to antibiotic prescription due to added medication risk [1] | **Effectiveness:**   - 160/425 (38%) reported suitability of VC better or the same for prescription of antibiotics [1] |
|  | - *Prescription of new medication via Telehealth rated lower in comparison to FTFC:* In comparison to FTFC, prescription of new medications via VC rated lower [1] | - Prescription of new medications via VC were rated lower than FTFC, with 167/429 (40%) rating it “better or same” than FTFC [1] |
|  | **Experience:**   - N/A visit specific evidence | **Experience:**   - N/A visit specific evidence |
|  | **Financial Impact/Cost:**   - N/A visit specific evidence | **Financial Impact/Cost:**   - N/A visit specific evidence |
| **Post-test results follow-up** | **Access to Care:**   - N/A visit specific evidence | **Access to Care:**   - N/A visit specific evidence |
|  | **Effectiveness:**   - N/A visit specific evidence | **Effectiveness:**   - N/A visit specific evidence |
|  | **Experience:**   - N/A visit specific evidence | **Experience:**   - N/A visit specific evidence |
|  | **Financial Impact/Cost:**   - N/A visit specific evidence | **Financial Impact/Cost:**   - N/A visit specific evidence |
| **Post-discharge follow-up** | **Access to Care:**   - *Harder to conduct consultations remotely with team-based care common with post-discharge patients:* post-discharge after surgery/procedure discussions often involves third party members that cannot always be made available [23] | **Access to Care:**   - “Sometimes we involve the nurses to give them the plan, but they are not doing telephone consultations” P4 [23] |
|  | **Effectiveness:**   - *Lower suitability rating of Telehealth consultation in comparison to FTF when surveyed:* Poor suitability response rates of visit type in comparison to FTF due to lack of physical presence and complex patient presentations [1] | **Effectiveness:**   - 30/79 (38%) clinician responses for the suitability of VC for hospitalisation or acute referral being “better” or “same”, indicating it is quite inadequate for any surgery or related procedural concerns [1] |
|  | - *Physical presence often required post-discharge for physical examinations:* Physical presence is often required in post-surgery or procedure discussions, which is not amendable to Telehealth [18] | - N/A visit specific evidence |
|  | - *Difficult to share documentation from multiple team members involved in post-discharge care via Telehealth (e.g., surgeons, nurses, discharge-clinic doctors):* Concerns surrounding the inability of sharing necessary post-discharge for ante-natal care and the consistency in Telehealth documentation [23] | - “There is no clear format what we should do but sent from DGHS what they want, for example; the number of consultations for NCD and ANC (ante-natal care) patients, just like a statistic.” P3, [23] |
|  | **Experience:**   - *Telehealth consultations are shorter, reducing the ability to gather information (e.g., patient concerns and further patient history):* Telehealth consultations tend to be shorter, which does not provide patients ample time to raise concerns [22] | **Experience:**   - “The conversation[s] seemed a little bit shorter. A patient in clinic . . . there are moments where there’s space for more conversation about not necessarily medical [problems] but just to get to know the patient a little better” (P15) [22] |
|  | **Financial Impact/Cost:**   - N/A visit specific evidence | **Financial Impact/Cost:**   - N/A visit specific evidence |
