# Supplementary material for: Visit Types in Primary Care With Telehealth Use During the COVID-19 Pandemic: Systematic Review
Source: JMIR Med Inform. 2022 Nov 28;10(11):e40469. doi: 10.2196/40469 (PMC9745650; doi:10.2196/40469)
Supplement: Multimedia Appendix 9 [file medinform_v10i11e40469_app9.docx]

# Appendix 9. Supporting Evidence of Benefit and Drawback of using Telehealth in Primary care during COVID-19 according to outcomes of the NQF Framework

## Access to Care

## Table 9A. Supporting Evidence of Benefit and Drawback Findings of Telehealth to ‘Access to Care’ Outcome Factor

| ***Benefit Findings of Telehealth*** | ***Drawback Findings of Telehealth*** |
| --- | --- |
| **Primary Care Clinician Perspective:** |  |
| - *Greater consultation rates with Telehealth* [25, 26] - *Improving completion rates with Telehealth:* [23] - *Improved perception of patients feeling satisfied with reduced wait times:*   “The patient’s time is also of value in my opinion - no less than ours, There’s no doubt that for every minute we invest, they save half an hour of driving to the clinic and finding parking.” (1) [26] | - *Minimal technical support available:* Technical problems identified to be frequent during sessions   The GPs experienced that some patients did not have the needed technical competencies or equipment. Some also had difficulties sitting correctly in front of the camera or handling it when they had to show things on their body. Here, the GPs also mentioned the work involved in informing patients about the technical issues before a video consultation. [9]   - *Harder to address language or cognition barriers* [32] - *Concerning technical issues:*   The quality of the video camera and the sharpness of the image, if you want to observe, for example a rash or swelling, it requires the patient being able to get the camera angled in the right place and that the light and the distance to what we want to see is okay […]. It is also preferable to use a Wi-Fi connection. A 3G network pixelates it and makes it blurry. It also means that some contrasts and sharpness are lost, and then you cannot really use it.” (GP4, interview 1) [9] |
| **Patient Perspective** |  |
| - *Limited technical support required for patients:*   I Googled, as they so popularly call it, and then I came across (name of telemedicine consultation provider). I thoughtthatIcouldtryandsee how itworked.I downloaded the app on my computer; it was really easy. I was impressed by how fast it was, and that this procedure was so effective. IP4, male, 73 years [23].   - *Reduced travel time* [31, 24] - *Reduced time spent at the clinic finding parking or waiting for appointment time:*   “No need to waste my time - either on the road or in line. I come in[to the room] a minute before [the video/phone call], [it’s] very convenient.” (1)“If I go to see the PCP [physically], it takes a few hours… but, if I see the PCP via a video call, it only takes as long as the conversation itself.” (2) [26]  “Why leave the house if you can get the same service and save the time spent waiting for the appointment?” (1) “I had a case with my daughter .... there was no appointment available, everything was booked several days ahead. Then I made a digital appointment for the same day. There were two available appointments: one in the afternoon and one in the evening. So, I made a digital appointment [video] for 7 pm that same day”. (1) [26]  ‘Usually [the consideration] is the queuing time. When I make an in-clinic appointment, I feel like I’m waiting for the PCP, [but when I make a virtual appointment, it’s as if] the PCP is waiting for me” (2). Queuing time before consultation played a very significant role here.” (1) [26]   - *Improved convenience:* [1, 22, 25, 26, 30, 31] - *Able to book consultations at convenient times:*   “I downloaded the app on my computer; it was really easy. I was impressed by how fast it was, and that his procedure was so effective.” IP, male, 73 years. [23]  “Digitally, I can contact at any time when I feel like it.” IP9, female, 47 years [23]  “Physical meetings with a PCP, from my personal experience, were always pushed into my schedule - either very early in the morning or late in the afternoon. Hours like ten o’clock, one o’clock or three o’clock, are just not realistic times for me, as someone who works full time. Digitally, [the appointment] can be anytime.” (2) If I need something not during his reception hours, I’ll call him, I’ll ask him, I’ll send him a WhatsApp message and ask if he can talk to me. Then, I’ll say okay…the call is recorded…it’s official.” (3) [26] | - *Isolating non-tech-savvy patients*“ [21, 22] - *Hesitant patients to use Telehealth:* Patient hesitancy causes concerns about not reaching patients in need of care (e.g., concerns of confidentiality, security, and required technology) [21] |
| **Both Primary Care Clinician and Patient Perspective** |  |
| - *Good technical quality experienced in majority of Telehealth consultations:* 3118/3475 (90%) of VC consultations had good technical quality for GPs and patients [1]. - *Enabling GPs and at-risk teenage patients to communicate easily:* Parents of teenage children mentioned it was easier to connect their child with a GP via a Telehealth consultation as they did not need to wait to go to the primary care centre [23] - Timely and more frequent access to care for at-risk patient groups due to convenience and limited wait times [1] | - Connectivity, audio, or visual issues impacting consultation quality [23] |

## Effectiveness

## ***Table 9B.*** ***Supporting Evidence of Benefit and Drawback findings of Telehealth to ‘Effectiveness’ Outcome Factor***

| ***Benefit Findings of Telehealth*** | ***Drawback Findings of Telehealth*** |
| --- | --- |
| **Primary Care Clinician Perspective** | |
| - *Improved access to medical records:*   95/169 (56%) of primary care paediatricians respondents reported full access to the child patient’s medical record in more than 75% of telehealth visits, while only 15/169 (9%) reported no access to the record during such visits [19]  “Ease in obtaining, recording, and transmitting necessary data” [19]   - *Existing healthcare system processes aids Telehealth establishment:* Facilitators of existing reimbursement mechanisms, access to user-friendly technology, and pre-existing staff relationships for reduced costs [25] - *Continual support from the same clinician not required for simple concerns:* Patients with administrative tasks, such as renewal of prescriptions, or more straightforward health problems such as skin rashes were deemed suitable to Telehealth due to no requirement for continual care and therefore patient-provider relationship [23] | - *Mixed views on the extent of effectiveness:* patient characteristics (e.g., age that would make them less suitable for Telehealth [26]   “If it’s for me, I’ll do it digitally; but if it’s for my child, I will come to the clinic.” (3) [26]   - *Missing indicators of serious adverse events a possibility with Telehealth:* In 514/3476 (15%) of the VCs, the GPs expressed concern that they might not have detected “potential signs of serious illness” [1]. - *Lacking physical examination:* The lack of opportunity to physically examine the patient was reported as a “major loss” or “some loss” in 884/3475 (25%) TC and 1232/3475 (36%) of VC [1] - *Reliant on clinicians taking on multiple roles in the practice:* Multiple roles required of a GP to manage telehealth consultations due to a lack of established infrastructure support.   “To overcome this lack of infrastructure, participants spoke about how they created purpose-built digital platforms or shouldered infrastructure costs themselves (for example, paying for software registrations or buying second mobile phones for business purposes). They further emphasised how improving this infrastructure might enable them to provide higher quality care:  'If we could improve the quality of the experience, to get it closer to that face to face; I think technology, education, and access to the equipment would bridge that gap.' (GP71) [9]   - *Poorer diagnostic ability:* Not suited to complex symptom presentations that require physical examinations (e.g., chest pain, stomach pain, potential new cancer) [1] |
| **Patient Perspective** | |
| - *Improving patient’s ability to self-manage their health conditions due to increased patient monitoring*: Telehealth enables a patient’s ability to self-manage and adhere to primary care provider advice due to more frequent meetings and discussions about health concerns [1] - *Appropriate ability of patients to self-assess suitability of Telehealth to their health concern:*   “Of course, you can’t use telemedicine consultations for everything, but for the things that I have used it for, it has worked very well. Yes, it [telemedicine consultation] works better for me than conventional primary care visits.” IP9, female 47 years [23]  “I have chosen the type of errands that I think can work and are suitable for this type of service.” IP5, female, 46 years [23] | - *Raised concerns of how to protect confidentiality due to Telehealth setting:* Sometimes people are not comfortable discussing health issues from within their homes (lack of privacy, unsafe environment to discuss concerns etc.) (S: F, 18–24) [23] - *Inevitable FTF consultations of concern: “*I had called because I was unsure what to do when my stomach pain was so bad, and I didn’t know why. Then the physician told me to go the primary care centre.” IPI, female, 18 years [23] |
| **Both Primary Care Clinician and Patient Perspective** | |
| - *Common health concerns suitable to Telehealth:* “The three most common diagnoses found in the register data were infections, dermatological complaints, and the need for prescriptions,” which are deemed suitable for Telehealth in the absence of complex presentations (i.e., comorbidity) [23] |  |

## Experience

## ***Table 9C.*** ***Supporting Evidence of Benefit and Drawback findings of Telehealth to ‘Experience’ Outcome Factor***

| ***Benefit findings of Telehealth*** | ***Drawback findings of Telehealth*** |
| --- | --- |
| **Primary Care Clinician Perspective** | |
| - *Improved work-life balance* [33] - *Satisfied in perceiving their patients to be more relaxed in Telehealth setting*s [32] | - *Concerned about literacy and language barriers with patients* [23] - *Lacking in stimulating work for clinicians* [9]   “You miss that tactile, therapeutic sort of presence, and you miss the sense of what, that you sort of get face to face.' (GP71) [9] |
| **Patient Perspective** | |
| - *Satisfying patient expectations:* High reported satisfaction across different telemedicine modes, with 42/46 (91%) for VC and 390/454 (86%) for TC, respectively [22] - *Willing patients to use Telehealth again:* 151/394 (38.3%) patients were willing to use a TC/VC again [21] - *Satisfying patients in making them feel like their health was well managed via Telehealth* [24, 31] | - *Lacking in establishing patient-provider relationship:* [23]   “The negative side of using telemedicine consultations is that they do not know who I am, and this makes the meeting less personal. There is no real contact and no follow-up, so it may be better to use conventional primary care when there is a need for that. IP5, female 46 years” [23]   - *Lacking in feeling attended to when patients have additional concerns adequately: “*I opened up a discussion about my stress and the life I live. There was an opening for being able to talk about it, but I experienced nil response. It was not even noticed; she prescribed the same medicine, and I never met with her again.” I14, male, 43 years [23] - *Impersonal:* “The negative side of using telemedicine consultations is that they do not know who I am, and this makes the meeting less personal. There is no real contact and no follow-up, so it may be better to use conventional primary care when there is a need for that.” IP5, female 46 years [23] |
| **Both Primary Care Clinician and Patient Perspective** | |
| - *Lower risk of infection transmission:* Remote consultations reduce the risk of infection transmission from diseases like COVID-19 [1, 23, 26]   “…And the [idea of the risk of] infection, which hangs over the clinics nowadays. It’s impossible to know what you might catch – that’s definitely one of the considerations.” (1) Whenever I come to the clinic, there are lots of people there - coughing, sneezing. Why do I need to get infected?”(3) [26]  “Now with COVID-19…I’m from an area that’s highly infected, so people try not to come if they can. So, I think [telemedicine] is a good tool.”(4) [26]   - *Positive patient-provider relationship* [32] | - *Lacking physical examinations:* Dissatisfaction with no physical examinations due to lack of certainty in diagnosis (e.g., respiratory concerns that require auscultation examination) for both clinicians and patients [9, 24, 33] |

## Financial Impact/Cost

## ***Table 9D.*** ***Supporting Evidence of Benefit and Drawback findings of Telehealth to ‘Financial Impact/Cost’ Outcome Factor***

| ***Benefit findings of Telehealth*** | ***Drawback findings of Telehealth*** |
| --- | --- |
| **Primary Care Clinician Perspective** | |
| - *Existing infrastructure processes reduce costs:* Reimbursement models (e.g., Medicare) available for clinicians [9, 24]   'I have used telehealth before COVID came for really remote patients, and often that was not funded by Medicare. They funded it themselves. COVID was a real relief, because those patients had suffered a lot from their remoteness and were now able to get help.' (GP40) [9].   - *Cost-effective due to reduced running costs:* Cost-effective due to reduced running costs (e.g., fewer in-person clinic single-use medical products or infrastructure-related costs) [9, 22, 31] | - *Problematic issues of reimbursement not yet determined:* Reimbursement models for long-term funding not determined globally [25] |
| **Patient Perspective** | |
| - *Willing patients to pay for Telehealth consultations:*   Patients willing to pay for Telehealth consultations either partially or in full [22]   - *Appropriate Telehealth consultation charges felt by some patients (inclusive of healthcare supported reimbursement)* [34] | - *Inappropriate Telehealth consultation charges felt by some patients:* “I was shocked I was charged $56.50 for a phone consult that lasted 10 minutes and did not include an examination of the affected area.” (S: F,45–54) [22] - *Lacking all patients to pay the full Telehealth consultation cost:* [32] - *Potential to be exploited by profitable companies:* “It is 2 minutes, and then you have prescription. […] it feels quite money driven if I am to be honest. So, there is a big financial gain in having a fast service because then you can feed off as many patients as possible.” IP14, male 43 years [23] |
| **Both Primary Care Clinician and Patient Perspective** | |
| - *Suitable Medicare support:* Pre-existing temporary COVID-19 Medicare items to cover the costs of Telehealth consultations [24, 25] - *Saving costs using Telehealth:* “Some users reasoned that telemedicine services were a way to off-load conventional primary care to favour patients with more complex health care needs. Therefore, there are potential savings on human and environmental resources, contributing to healthy and sustainable living” 23] | - N/A |
